# Synthesis and Cytotoxic Activity on Human Cancer Cells of Novel Isoquinolinequinone–Amino Acid Derivatives

**DOI:** 10.3390/molecules21091199

**Published:** 2016-09-08

**Authors:** Jaime A. Valderrama, Virginia Delgado, Sandra Sepúlveda, Julio Benites, Cristina Theoduloz, Pedro Buc Calderon, Giulio G. Muccioli

**Affiliations:** 1 Facultad de Ciencias de la Salud, Universidad Arturo Prat, Casilla 121, Iquique 1100000, Chile; sandra.sepulveda.ordenes@gmail.com (S.S.); juliob@unap.cl (J.B.); pedro.buccalderon@uclouvain.be (P.B.C.); 2 Instituto de Ciencias Exactas y Naturales Universidad Arturo Prat, Casilla 121, Iquique 1100000, Chile; 3 Facultad de Química, Pontificia Universidad Católica de Chile, Casilla 306, Santiago 26094411, Chile; vcdelgad@uc.cl; 4 Facultad de Ciencias de la Salud, Universidad de Talca, Talca 3460000, Chile; ctheodul@utalca.cl; 5 Research Group of Toxicology and Cancer Biology, Louvain Drug Research Institute, Université Catholique de Louvain, 73 Avenue E. Mounier, GTOX 7309, Brussels 1200, Belgium; 6 Bioanalysis and Pharmacology of Bioactive Lipids Laboratory, Louvain Drug Research Institute, Université Catholique de Louvain, 72 Avenue E. Mounier, BPBL 7201, Brussels 1200, Belgium; giulio.muccioli@uclouvain.be

**Keywords:** α-amino acid methyl ester, isoquinolinequinones, tumor cell lines, cytotoxic activity

## Abstract

A variety of aminoisoquinoline-5,8-quinones bearing α-amino acids moieties were synthesized from 3-methyl-4-methoxycarbonylisoquinoline-5,8-quinone and diverse l- and d-α-amino acid methyl esters. The members of the series were evaluated for their cytotoxic activity against normal and cancer cell lines by using the (3-(4,5-dimethylthiazol-2-yl)-2,5-diphenyltetrazolium bromide) (MTT) assay. From the current investigation, structure–activity relationships demonstrate that the location and structure of the amino acid fragment plays a significant role in the cytotoxic effects. Moderate to high cytotoxic activity was observed and four members, derived from l-alanine, l-leucine, l-phenylalanine, and d-phenylalanine, were selected as promising compounds by their IC_50_ ranging from 0.5 to 6.25 μM and also by their good selectivity indexes (≥2.24).

## 1. Introduction

Quinones (anthraquinones, naphthoquinones, and heterocyclic quinones) are important naturally occurring substances that are widely distributed in nature [1] and are known by their various physiological activities as antibiotics and anticancer agents. They form the second large class of antitumor agents approved for clinical use and several other antitumor members are currently in different stages of clinical and preclinical development. Their antitumor activity has been currently explained by their ability to generate reactive oxygen species (ROS) via a redox cycling process, by DNA intercalation and by covalent binding to essential proteins and/or DNA [2,3,4,5,6].

In this context, the therapeutic index of most anticancer therapeutic drugs is limited by the magnitude of the relative differences in the biological sensitivities of cancer cells and normal cells. Indeed, most anticancer drugs have a severe toxicity toward normal cells. Thus, the greatest challenge in this area is how to target the active species (e.g., quinones) and their cytotoxicity so that they will distinguish between normal healthy cells and damaged cells. Therefore, there is still an urgent need for new and more effective drugs and innovative drug design strategies.

**Figure 1:**
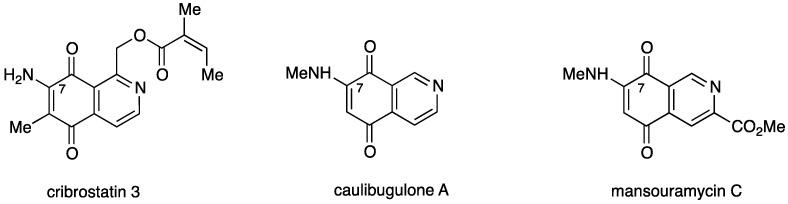
Structures of naturally occurring 7-aminoisoquinolinequinones with anticancer activity.

Among the broad variety of *N*-heterocyclic quinones with anti-cancer activity are the naturally occurring 7-aminoisoquinolinequinones such as cribrostatin 3 [7], caulibugulone A [8], and mansouramycin C [9] (Figure 1).

Caulibugulone A was found to be a potent and selective inhibitor of the Cdc25B phosphatase, a potential human oncogene [10], by oxidizing and inactivating the catalytic cysteine of the enzyme through redox cycling and production of ROS [11,12]. In a previous work we reported that a series of synthetic amino- and alkylaminoisoquinolinequinones, structurally related to mansouramycin C, display significant in vitro cytotoxic activity on gastric adenocarcinoma, lung cancer, and human bladder carcinoma, but low selective index referred to human lung fibroblasts [13].

In order to add new members to this promising chemical series to define the critical structural elements required for potency and selectivity, we are interested in evaluating substances containing biologically relevant nitrogen substituents such as natural α-amino acids. Concerning the design of isoquinolinequinone-α-amino acid derivatives, it was based on the cytotoxic activity of α-amino acid-containing natural occurring 1,4-benzoquinones [14,15,16], 1,4-naphthoquinone [17,18,19], and 9,10-anthraquinone [20].

## 2. Results and Discussion

### 2.1. Chemistry

The preparation of the isoquinolinequinone-α-amino acid derivatives was carried out by reaction of the known isoquinolinequinone **1** [13] with a variety of α-amino acid methyl esters in ethanol, under aerobic conditions at room temperature (Table 1). In all cases the reaction gives a mixture of the respective regioisomers, as was observed by TLC and ^1^H-NMR. Pure samples were isolated by column chromatography of the respective mixtures, but efforts to isolate the minor regioisomers **2b**, **5b**, **9b**, and **11b**, were unsuccessful. The structures of the new compounds were fully established by IR, ^1^H-, ^13^C-NMR, and high resolution mass spectrometry (HRMS).

The results show that the amination reaction of quinone **1** proceeds with regioselective preference to give the 7-substituted isomer as the main product. The control of the regioselectivity could be ascribed to the electron-withdrawing effect of the heterocyclic nitrogen atom in **1**, which makes the C-5 carbonyl group more electron deficient, directing the nucleophilic attack of the amine to C-7.

It is worth mentioning that since there is no doubt of the inversion of configuration at chiral center, the absolute configuration of the l- and d-α-amino acid methyl ester is reflected in the corresponding aminoisoquinolinequinones. The optical rotations of the products could not be determined due to the darkening of solutions.

A number of bromine derivatives of the aminoisoquinolinequinones were prepared by reaction of the corresponding precursors with *N*-bromosuccinimide in methanol at room temperature (Table 2).

### 2.2. In Vitro Cytotoxic Activity of Isoquinolinequinone-Amino Acid Derivatives on Cancer Cell Lines

The series of isoquinolinequinone amino ester conjugates (Table 1) and the bromine derivatives (Table 2) were evaluated for their in vitro cytotoxic activities against normal human lung fibroblast (MRC-5) and three human cancer cells lines: human gastric adenocarcinoma (AGS), human lung cancer (SK-MES-1), and human bladder carcinoma (J82), in 72 h drug exposure assays. The cytotoxic activities of the new compounds were measured using conventional microculture tetrazolium reduction assays [21]. The cytotoxic activities of each of the quinones are expressed in terms of IC_50_. Etoposide, a clinically used anticancer agent, was taken as a positive control. The cytotoxic activity data are summarized in Table 3 and Table 4.

The structure–activity relationship (SAR) will be firstly analyzed for aminoisoquinolinequinones **2a**–**11a** and **3b**, **4b**, **6b**–**8b**, and **10b**. The data in Table 3 reveals that, in general, moderate to high cytotoxic activity was observed for the members of the series with IC_50_ values ranging from 0.58 to 15.43 μM, while the positive control, etoposide, exhibits IC_50_ values in the range of 0.58 to 3.49 μM. A differential sensitivity to the aminoisoquinolinequinones was shown by the three different cell lines. The most sensitive were the gastric-derived AGS cells, while the least sensitive was the urogenital J82 cell line. It may be possible that the J82 cells, because they have a mutated form of p53, are more resistant against an oxidant agent, as has been shown for cisplatin treatment [22]. It may be observed that the location of the amino acid fragment at C-6 (regioisomer **b**) enhanced the cytotoxic effect as compared to its regioisomer **a**, where the nitrogen fragment is at C-7. Among the series, compounds **3b**, **4b**, **8b**, and **10b** exhibited higher cytotoxic potencies (0.58–3.18 μM), comparable to that of etoposide (0.58–3.49 μM). Nevertheless, such increased activity was accompanied by a loss of selectivity.

Table 3 also shows the values of the Selective Index (SI), calculated as the ratio between IC_50_ values obtained for normal fibroblast cells and those IC_50_ values calculated for cancer cells. A high SI value (>2) of a compound gives a selective toxicity towards cancer cells. On the other hand, a compound with SI value <2 is considered to give general toxicity, i.e., it can also cause cytotoxicity on normal cells [23]. Based on this, compounds **2a**, **6a**, and **10a** exhibit high cytotoxic activity on cancer cells (mean IC_50_ values <4.0 μM) and selective effect on cancer cells (MSI ≥ 2.24). Compound **10a**, derived from d-phenylalanine, appears as the most active compound (mean IC_50_ = 2.83 μM) and selective on cancer cells (mean SI = 2.63). It is noteworthy that compound **7a**, derived from l-tyrosine, is less potent and has a MSI ≤ 2 as compared to **6a**, derived from l-phenylalanine, suggesting that the insertion of a *p*-hydroxyl group in the amino acid fragment of the latter decreases the cytotoxicity against cancer cells. Inspection of the data in Table 3 indicates that the size of the amino acid fragment is not a determining factor in the resultant cytotoxic activity. Also, it is worth noting that the enantiomeric pairs **2a**/**9a**, **6a**/**10a**, and **8a**/**11a** exhibit differences in terms of their cytotoxic activity and selectivity index. These facts suggest that chiral recognition is probably involved in the mechanism of cytotoxic activity.

The cytotoxic properties of the bromine derivative series are summarized in Table 4. The data reveal that the bromine insertion at C-6 in precursors **2a** and **3a** (as in **12**, **13a**) and at C-7 in **3b** (as in **13b**) does not induce significant changes in the cytotoxic activity and selectivity. Now, by comparing them to each respective precursor, bromine substitution in compounds **12**, **13a**, and **15** enhances their cytotoxicity activity but they lose the selectivity shown by their precursors **2a**, **3a**, and **6a**, respectively. It is noteworthy that the bromine insertion at C-6 in compound **4a** (as in **14**), dramatically influences the cytotoxic activity of the aminoisoquinolinequinone scaffold. In fact, compound **14** exhibits selective and potent cytotoxic activity on lung and bladder cancer cells (MSI > 45) but not on normal fibroblasts (SI > 100) or gastric cancer cells. This compound is a special case: indeed, it has shown extremely low activity against AGS cells, the cancer cell line of highest sensitivity, as well as against healthy fibroblasts (MRC-5). We do not have any explanation for such low cytotoxic activities.

In order to gain insight into the high selectivity of compound **14** on MRC-5 cells, we explored further its cytotoxic properties in other lines including T24 (bladder), DU-145 (prostate), MCF-7 (breast), and normal human fibroblasts (AG 1523). In these assays the anticancer drugs tamoxifen and 5-fluorouracil were used as positive controls (Table 5). The results show that compound **14** induces a high cytotoxic activity in cancer cells (IC_50_ 0.50–0.67 μM) while it shows rather low activity in healthy fibroblasts (AG 1523). These results provide support to the absence of effect of **14** on fibroblasts (MRC-5) at IC_50_ level <100 μM, thus indicating that compound **14** exhibits a fairly high selective index.

## 3. Experimental Section

### 3.1. General Information

All the solvents and reagents were purchased from different companies such as Aldrich (St. Louis, MO, USA) and Merck (Darmstadt, Germany) and were used as supplied. Melting points were determined on a Stuart Scientific SMP3 (Staffordshire, UK) apparatus and are uncorrected. The IR spectra were recorded on an FT IR Bruker spectrophotometer, model Vector 22 (Bruker, Rheinstetten, Germany), using KBr disks, and the wave numbers are given in cm^−1^. ^1^H-NMR spectra were recorded on a Bruker Avance-400 instrument (Bruker) in deuterochloroform (CDCl_3_). ^13^C-NMR spectra were obtained in CDCl_3_ at 100 MHz. Bidimensional NMR techniques and distortionless enhancement by polarisation transfer (DEPT) were used for signal assignment. Chemical shifts are expressed in ppm downfield relative to tetramethylsilane. Data for ^1^H-NMR spectra are reported as follows: s = singlet, d =doublet, m = multiplet and the coupling constants (*J*) in Hz. The HRMS spectra were obtained on a Thermo Finnigan spectrometer, model MAT 95XP and LTQ-Orbitrap mass spectrometer (Thermo-Fisher Scientific, Waltham, MA, USA) with the analysis performed using an atmospheric-pressure chemical ionization (APCI) source operated in positive mode. Silica gel Merck 60 (70–230 mesh, from Merck) was used for preparative column chromatography and TLC aluminum foil 60F_254_ for analytical thin layer chromatography (TLC). Isoquinolinequinone **1** was prepared according to the procedure previously reported [13].

### 3.2. Synthesis

#### 3.2.1. Preparation of Isoquinolinequinone-Amino Acid Derivatives. General Procedure

Suspensions of isoquinolinequinone **1** (1 equiv.), l- or d*-*α-amino acid methyl esters hydrochloride (2 equiv.) and NaOAc (2 equiv.) in ethanol (15 mL) were left with stirring at room temperature (rt) after completion of the reaction as indicated by TLC. The solvent was removed under reduced pressure and the residue was purified by chromatography over silica gel (90:10 CH_2_Cl_2_/EtOAc) to yield mixtures of regioisomers, in ratios determined by ^1^H-NMR in CDCl_3_. Further column chromatography of the mixture of isomers over silica gel (CH_2_Cl_2_), provided pure samples of the regioisomers.

*Methyl 6- and 7-(1-methoxy-1-oxopropan-2-ylamino)-3-methyl-5,8-dioxo-5,8-dihydroisoquinoline-4-carboxylate* (**2a**, **2b**). The mixture of regioisomers was prepared from **1** (200 mg, 0.87 mmol) and l-alanine methyl ester hydrochloride (2 h). Compound **2a** (less polar, 104 mg, 0.31 mmol, 53%): orange solid, mp: 141–143 °C; IR ν_max_: 3370 and 3324 (N-H), 2954 and 2930 (C-H), 1745 (C=O ester); 1687 (C=O quinone); ^1^H-NMR (400 MHz, CDCl_3_): δ 1.57 (d, 3H, *J* = 6.8 Hz, 12-Me), 2.65 (s, 3H, 3-Me), 3.81 (s, 3H, 12-CO_2_Me), 4.03 (s, 3H, 4-CO_2_Me), 4.13 (m, 1H, 12-H), 5.70 (s, 1H, 6-H), 6.46 (d, 1H, *J* = 7.2 Hz, NH), 9.19 (s, 1H, 1-H); ^13^C-NMR (100 MHz, CDCl_3_): δ 17.6 (CH_3_, 12-CH_3_), 23.1 (CH_3_, C-9), 50.7 (CH, C-12), 53.1 (CH_3_, C-10), 53.2 (CH_3_, C-14), 102.4 (CH, C-6), 122.0 (C, C-8a), 126.2 (C, C-4), 135.8 (C, C-4a), 146.4 (C, C-7), 148.3 (CH, C-1), 163.0 (C, C-3), 168.8 (C, C-11), 171.7 (C, C-13), 180.1 (C, C-5), 180.7 (C, C-8); HRMS (M^+^): *m*/*z* calcd. for C_16_H_16_N_2_O_6_: 332.10084; found: 332.10707. Attempts to isolate a pure sample of **2b** were unsuccessful.

*Methyl 6- and 7-(1-methoxy-3-methyl-1-oxobutan-2-ylamino)-3-methyl-5,8-dioxo-5,8-dihydro-isoquinoline-4-carboxylate* (**3a**, **3b**). The mixture of regioisomers was prepared from **1** (200 mg, 0.87 mmol) and l-valine methyl ester hydrochloride (2 h). Compound **3a** (less polar, 128 mg, 0.36 mmol, 58%): orange solid, mp: 91.5–93 °C; IR ν_max_: 3368 (N-H), 2970 and 2930 (C-H), 1734 (C=O ester); 1686 (C=O quinone); ^1^H-NMR (400 MHz, CDCl_3_): δ 1.02 (d, 3H, *J* = 6.8 Hz, 12-C-Me), 1.08 (d, 3H, *J* = 6.8 Hz, 12-C-Me), 2.31 (m, 1H, 12-C-CH), 2.65 (s, 3H, 3-Me), 3.80 (s, 3H, 12-CO_2_Me), 3.92 (m, 1H, 12-H), 4.03 (s, 3H, 4-CO_2_Me), 5.73 (s, 1H, 6-H), 6.48 (d, 1H, *J* = 8.0 Hz, NH), 9.19 (s, 1H, 1-H); ^13^C-NMR (100 MHz, CDCl_3_): δ 18.4 (CH_3_, 12-CHCH_3_), 18.8 (CH_3_, 12-CHCH_3_), 22.9 (CH_3_, C-9), 31.2 (CH, 12-CH), 52.6 (CH_3_, C-14), 53.1 (CH_3_, C-10), 60.5 (CH, C-12), 102.2 (CH, C-6), 121.8 (C, C-8a), 126.0 (C, C-4), 135.6 (C, C-4a), 147.0 (C, C-7), 148.1 (CH, C-1), 162.8 (C, C-3), 168.6 (C, C-11), 170.5 (C, C-13), 180.0 (C, C-5), 180.5 (C, C-8); HRMS (M^+^): *m*/*z* calcd. for C_18_H_20_N_2_O_6_: 360.13214; found: 360.13813.

Compound **3b** (more polar, 15 mg, 0.04 mmol, 7%): orange solid, mp: 81.5–83 °C; IR ν_max_: 3375 (N-H), 2959 and 2931 (C-H), 1739 (C=O ester); 1686 (C=O quinone); ^1^H-NMR (400 MHz, CDCl_3_): δ 1.01 (d, 3H, *J* = 6.8 Hz, 12-C-Me), 1.06 (d, 3H, *J* = 6.8 Hz, 12-C-Me), 2.29 (m, 1H, 12-C-CH), 2.66 (s, 3H, 3-Me), 3.80 (s, 3H, 12-CO_2_Me), 3.89 (m, 1H, 12-H), 4.06 (s, 3H, 4-CO_2_Me), 5.72 (s, 1H, 7-H), 6.22 (d, 1H, *J* = 8.4 Hz, NH), 9.28 (s, 1H, 1-H); ^13^C-NMR (100 MHz, CDCl_3_): δ 18.5 (CH_3_, 12-CHCH_3_), 18.9 (CH_3_, 12-CHCH_3_), 22.7 (CH_3_, C-9), 31.3 (CH, 12-CH), 52.7 (CH_3_, C-14), 53.4 (CH_3_, C-10), 60.6 (CH, C-12), 102.3 (CH, C-7), 122.7 (C, C-8a), 125.1 (C, C-4), 132.3 (C, C-4a), 146.9 (C, C-6), 148.8 (CH, C-1), 160.3 (C, C-3), 168.4 (C, C-11), 170.7 (C, C-13), 181.0 (C, C-5), 181.8 (C, C-8); HRMS (M^+^): *m*/*z* calcd. for C_18_H_20_N_2_O_6_: 360.13214; found: 360.13788.

*Methyl 6- and 7-(1-methoxy-4-methyl-1-oxopentan-2-ylamino)-3-methyl-5,8-dioxo-5,8-dihydroisoquinoline-4-carboxylate* (**4a**, **4b**). The mixture of regioisomers was prepared from **1** (200 mg, 0.87 mmol) and l-leucine methyl ester hydrochloride (2 h). Compound **4a** (less polar, 132 mg, 0.35 mmol, 56%): orange solid, mp: 90.5–92.5 °C; IR ν_max_: 3371 and 3326 (N-H), 2956 and 2929 (C-H), 1749 (C=O ester); 1693 (C=O quinone); ^1^H-NMR (400 MHz, CDCl_3_): δ 0.85 (d, 3H, *J* = 6.4 Hz, 12-CH_2_-C-Me), 0.91 (d, 3H, *J* = 6.4 Hz, 12-CH_2_-C-Me), 1.68 (m, 1H, 12-CH_2_-CH), 1.72 (m, 2H, 12-CH_2_), 2.57 (s, 3H, 3-Me), 3.70 (s, 3H, 12-CO_2_Me), 3.95 (s, 4H, 4-CO_2_Me and 12-H), 5.64 (s, 1H, 6-H), 6.26 (d, 1H, *J* = 8.0 Hz, NH), 9.10 (s, 1H, 1-H); ^13^C-NMR (100 MHz, CDCl_3_): δ 22.0 (CH_3_, 2-CHCH_3_), 22.5 (CH_3_, 12-CHCH_3_), 23.0 (CH_3_, C-9), 25.0 (CH, 12-CH_2_CH), 40.7 (CH, 12-CH), 52.8 (CH_3_, C-14), 53.1 (CH_3_, C-10), 53.6 (CH, C-12), 102.2 (CH, C-6), 121.8 (C, C-8a), 126.0 (C, C-4), 135.6 (C, C-4a), 146.8 (C, C-7), 148.1 (CH, C-1), 162.9 (C, C-3), 168.6 (C, C-11), 171.5 (C, C-13), 180.0 (C, C-5), 180.6 (C, C-8); HRMS (M^+^): *m*/*z* calcd. for C_19_H_22_N_2_O_6_: 374.14779; found: 374.15351.

Compound **4b** (more polar, 17 mg, 0.04 mmol, 7%): orange solid, mp: 58.5–60.5 °C; IR ν_max_: 3367 (N-H), 2956 and 2928 (C-H), 1739 (C=O ester); 1687 (C=O quinone); ^1^H-NMR (400 MHz, CDCl_3_): δ 0.93 (d, 3H, *J* = 6.0 Hz, 12-CH_2_-C-Me), 0.99 (d, 3H, *J* = 6.0 Hz, 12-CH_2_-C-Me), 1.76 (m, 3H, 12-CH_2_ and 12-CH_2_-CH), 2.66 (s, 3H, 3-Me), 3.78 (s, 3H, 12-CO_2_Me), 4.03 (s, 4H, 4-CO_2_Me and 12-H), 5.72 (s, 1H, 7-H), 6.01 (d, 1H, *J* = 8.0 Hz, NH), 9.28 (s, 1H, 1-H); ^13^C-NMR (100 MHz, CDCl_3_): δ 22.1 (CH_3_, C-9), 22.7 (2CH_3_, 12-CHCH_3_), 25.0 (CH, 12-CH_2_CH), 40.9 (CH, 12-CH), 53.0 (CH_3_, C-14), 53.4 (CH_3_, C-10), 53.7 (CH, C-12), 102.4 (CH, C-7), 122.7 (C, C-8a), 125.0 (C, C-4), 132.3 (C, C-4a), 146.6 (C, C-6), 148.8 (CH, C-1), 160.4 (C, C-3), 168.4 (C, C-11), 171.7 (C, C-13), 181.0 (C, C-5), 181.9 (C, C-8); HRMS (M^+^): *m*/*z* calcd. for C_19_H_22_N_2_O_6_: 374.14779; found: 374.15335.

*Methyl 6- and 7-(1-methoxy-4-(methylthio)-1-oxobutan-2-ylamino)-3-methyl-5,8-dioxo-5,8-dihydroisoquinoline-4-carboxylate* (**5a**, **5b**). The mixture of regioisomers was prepared from **1** (200 mg, 0.87 mmol) and l-methionine methyl ester hydrochloride (1 h 30 min). Compound **5a** (less polar, 125 mg, 0.32 mmol, 52%): orange solid, mp: 66.5–68 °C; IR ν_max_: 3353 (N-H), 2951 and 2918 (C-H), 1738 (C=O ester); 1681 (C=O quinone); ^1^H-NMR (400 MHz, CDCl_3_): δ 2.11 (s, 3H, Me-S), 2.23 (m, 2H, S-CH_2_), 2.60 (m, 2H, 12-CH_2_), 2.66 (s, 3H, 3-Me), 3.82 (s, 3H, 12-CO_2_Me), 4.03 (s, 3H, 4-CO_2_Me), 4.33 (m, 1H, 12-H), 5.830 (s, 1H, 6-H), 6.59 (d, 1H, *J* = 8.4 Hz, NH), 9.19 (s, 1H, 1-H); ^13^C-NMR (100 MHz, CDCl_3_): δ 15.5 (CH_3_, S-CH_3_), 22.9 (CH_3_, C-9), 29.8 (CH_2_, 12-CH_2_), 30.6 (CH_2_, S-CH_2_), 53.0 (CH_3_, C-14), 53.1 (CH_3_, C-10), 53.6 (CH, C-12), 102.4 (CH, C-6), 121.8 (C, C-8a), 126.0 (C, C-4), 135.5 (C, C-4a), 146.7 (C, C-7), 148.0 (CH, C-1), 162.8 (C, C-3), 168.5 (C, C-11), 170.8 (C, C-13), 180.0 (C, C-5), 180.5 (C, C-8); HRMS (M^+^): *m*/*z* calcd. for C_18_H_2__0_N_2_O_6_S: 392.10421; found: 392.10987. Attempts to isolate a pure sample of **5b** were unsuccessful.

*Methyl 6- and 7-(1-methoxy-1-oxo-3-phenylpropan-2-ylamino)-3-methyl-5,8-dioxo-5,8-dihydroisoquinoline-4-carboxylate* (**6a**, **6b**). The mixture of regioisomers was prepared from **1** (200 mg, 0.87 mmol) and l-phenylalanine methyl ester hydrochloride (2 h 30 min).Compound **6a** (less polar, 149 mg, 0.37 mmol, 60%): orange solid, mp: 118–119.5 °C; IR ν_max_: 3353 and 3287 (N-H), 2947 and 2927 (C-H), 1735 (C=O ester); 1685 (C=O quinone); ^1^H-NMR (400 MHz, CDCl_3_): δ 2.64 (s, 3H, 3-Me), 3.17 (dd, 1H, *J* = 6.4; 14 Hz, 12-CH_2_), 3.26 (dd, 1H, *J* = 5.6; 14 Hz, 12-CH_2_), 3.76 (s, 3H, 12-CO_2_Me), 4.02 (s, 3H, 4-CO_2_Me), 4.31 (m, 1H, 12-H), 5.68 (s, 1H, 6-H), 6.42 (d, 1H, *J* = 7.6 Hz, NH), 7.11 (m, 2H, arom), 7.28 (m, 3H, arom), 9.16 (s, 1H, 1-H); ^13^C-NMR (100 MHz, CDCl_3_): δ 23.0 (CH_3_, C-9), 37.5 (CH_2_, 12-CH_2_), 52.9 (CH_3_, C-14), 53.2 (CH_3_, C-10), 56.1 (CH, C-12), 102.5 (CH, C-6), 121.8 (C, C-8a), 126.1 (C, C-4), 127.8 (C, C-4’), 129.0 (2CH, C-2’ and C-6’), 129.1 (2CH, C-3’ and C-5’), 134.8 (C, 12-CH_2_, C-1’), 135.6 (C, C-4a), 146.4 (C, C-7), 148.2 (CH, C-1), 163.0 (C, C-3), 168.7 (C, C-11), 170.3 (C, C-13), 179.9 (C, C-8), 180.6 (C, C-5); HRMS (M^+^): *m*/*z* calcd. for C_22_H_20_N_2_O_6_: 408.13214; found: 408.13756.

Compound **6b** (more polar, 17 mg, 0.04 mmol, 7%): orange solid, mp: 72–73.5 °C; IR ν_max_: 3350 and 3284 (N-H), 2946 and 2929 (C-H), 1735 (C=O ester); 1684 (C=O quinone); ^1^H-NMR (400 MHz, CDCl_3_): δ 2.65 (s, 3H, 3-Me), 3.14 (dd, 1H, *J* = 6.8; 14 Hz, 12-CH_2_), 3.25 (dd, 1H, *J* = 5.6; 14 Hz, 12-CH_2_), 3.76 (s, 3H, 12-CO_2_Me), 4.04 (s, 3H, 4-CO_2_Me), 4.31 (m, 1H, 12-H), 5.67 (s, 1H, 7-H), 6.18 (d, 1H, *J* = 7.6 Hz, NH), 7.13 (m, 2H, arom), 7.29 (m, 3H, arom), 9.26 (s, 1H, 1-H); ^13^C-NMR (100 MHz, CDCl_3_): δ 22.7 (CH_3_, C-9), 37.7 (CH_2_, 12-CH_2_), 53.0 (CH_3_, C-14), 53.4 (CH_3_, C-10), 56.3 (CH, C-12), 102.6 (CH, C-7), 122.7 (C, C-8a), 125.1 (C, C-4), 127.8 (C, C-4’), 129.1 (2CH, C-2’ and C-6’), 129.2 (2CH, C-3’ and C-5’), 132.3 (C, C-4a), 134.8 (C, 12-CH_2_, C-1’), 146.3 (C, C-6), 148.8 (CH, C-1), 160.4 (C, C-3), 168.4 (C, C-11), 170.5 (C, C-13), 180.8 (C, C-5), 181.8 (C, C-8); HRMS (M^+^): *m*/*z* calcd. for C_22_H_20_N_2_O_6_: 408.13214; found: 408.13762.

*Methyl 6- and 7-(3-(4-hydroxyphenyl)-1-methoxy-1-oxopropan-2-ylamino)-3-methyl-5,8-dioxo-5,8-dihydroisoquinoline-4-carboxylate* (**7a**, **7b**). The mixture of regioisomers was prepared from **1** (200 mg, 0.87 mmol) and l-tyrosine methyl ester hydrochloride (1 h 40 min). Compound **7a** (less polar, 141 mg, 0.33 mmol, 56%): orange solid, mp: 93–94.5 °C; IR ν_max_: 3358 (N-H), 2953 and 2851 (C-H), 1738 (C=O ester); 1683 (C=O quinone); ^1^H-NMR (400 MHz, CDCl_3_): δ 2.63 (s, 3H, 3-Me), 3.06 (dd, 1H, *J* = 6.4; 14 Hz, 12-CH_2_), 3.14 (dd, 1H, *J* = 5.6; 14 Hz, 12-CH_2_), 3.75 (s, 3H, 12-CO_2_Me), 4.01 (s, 3H, 4-CO_2_Me), 4.26 (m, 1H, 12-H), 5.68 (s, 1H, 6-H), 6.49 (d, 1H, *J* = 8.0 Hz, NH), 6.71 (d, 2H, *J* = 8.4 Hz, arom), 6.91 (d, 2H, *J* = 8.4 Hz, arom), 7.75 (br s, 1H, OH), 9.12 (s, 1H, 1-H); ^13^C-NMR (100 MHz, CDCl_3_): δ 22.7 (CH_3_, C-9), 36.4 (CH_2_, 12-CH_2_), 52.9 (CH_3_, C-14), 53.3 (CH_3_, C-10), 56.2 (CH, C-12), 102.1 (CH, C-6), 116.0 (2CH, C-3’ and C-5’), 122.0 (C, C-8a), 125.9 (C, C-4), 126.2 (C, 12-CH_2_, C-1’), 130.2 (2CH, C-2’ and C-6’), 135.8 (C, C-4a), 146.6 (C, C-7), 147.9 (CH, C-1), 155.9 (CH, C4’-OH), 162.7 (C, C-3), 168.7 (C, C-11), 170.5 (C, C-13), 179.5 (C, C-8), 180.6 (C, C-5); HRMS (M^+^): *m*/*z* calcd. for C_22_H_20_N_2_O_7_: 424.12705; found: 424.13256.

Compound **7b** (more polar, 13 mg, 0.03 mmol, 5%): orange solid, mp: 76.5–78.5 °C; IR ν_max_: 3360 (N-H), 2953 and 2848 (C-H), 1739 (C=O ester); 1685 (C=O quinone); ^1^H-NMR (400 MHz, CDCl_3_): δ 2.65 (s, 3H, 3-Me), 3.09 (dd, 1H, *J* = 6.4; 14 Hz, 12-CH_2_), 3.16 (dd, 1H, *J* = 5.6; 14 Hz, 12-CH_2_), 3.76 (s, 3H, 12-CO_2_Me), 4.04 (s, 3H, 4-CO_2_Me), 4.25 (m, 1H, 12-H), 5.23 (br s, 1H, OH), 5.67 (s, 1H, 7-H), 6.19 (d, 1H, *J* = 7.6 Hz, NH), 6.76 (d, 2H, *J* = 8.4 Hz, arom), 6.97 (d, 2H, *J* = 8.4 Hz, arom), 9.27 (s, 1H, 1-H); ^13^C-NMR (100 MHz, CDCl_3_): δ 22.6 (CH_3_, C-9), 36.7 (CH_2_, 12-CH_2_), 52.9 (CH_3_, C-14), 53.35 (CH_3_, C-10), 56.2 (CH, C-12), 102.3 (CH, C-7), 115.9 (2CH, C-3’ and C-5’), 122.6 (C, C-8a), 125.3 (C, C-4), 126.3 (C, 12-CH_2_, C-1’), 130.3 (2CH, C-2’ and C-6’), 134.9 (C, C-4a), 146.6 (C, C-6), 147.4 (CH, C-1), 155.9 (CH, C4’-OH), 162.8 (C, C-3), 168.6 (C, C-11), 170.5 (C, C-13), 179.5 (C, C-8), 179.6 (C, C-5); HRMS (M^+^): *m*/*z* calcd. for C_22_H_20_N_2_O_7_: 424.12705; found: 424.13239.

*Methyl 6- and 7-(3-(1H-indol-3-yl)-1-methoxy-1-oxopropan-2-ylamino)-3-methyl-5,8-dioxo-5,8-dihydroisoquinoline-4-carboxylate* (**8a**, **8b**). The mixture of regioisomers was prepared from **1** (200 mg, 0.87 mmol) and l-tryptophan methyl ester hydrochloride (2 h). Compound **8a** (less polar, 214 mg, 0.48 mmol, 67%): orange solid, mp: 98.5–100 °C; IR ν_max_: 3360 (N-H), 2952 and 2927 (C-H), 1737 (C=O ester); 1681 (C=O quinone); ^1^H-NMR (400 MHz, CDCl_3_): δ 2.63 (s, 3H, 3-Me), 3.35 (dd, 1H, *J* = 6.4; 15 Hz, 12-CH_2_), 3.40 (dd, 1H, *J* = 5.2; 15 Hz, 12-CH_2_), 3.70 (s, 3H, 12-CO_2_Me), 4.01 (s, 3H, 4-CO_2_Me), 4.33 (m, 1H, 12-CH), 5.64 (s, 1H, 6-H), 6.47 (d, 1H, *J* = 7.6 Hz, NH), 6.98 (m, 1H, CH), 7.07 (m, 1H, arom), 7.13 (m, 1H, arom), 7.28 (d, 1H, *J* = 8.0 Hz, arom), 7.45 (d, 1H, *J* = 8.0 Hz, arom), 8.45 (br s, 1H, NH), 9.10 (s, 1H, 1-H); ^13^C-NMR (100 MHz, CDCl_3_): δ 23.0 (CH_3_, C-9), 27.5 (CH_2_, 12-CH_2_), 53.0 (CH_3_, C-14), 53.2 (CH_3_, C-10), 55.5 (CH, C-12), 102.2 (CH, C-6), 108.8 (C, 12-CH_2_C), 111.6 (CH, HN-CCH), 118.2 (CH, 12-CH_2_CCCH), 120.0 (CH, HN-CCHCH), 121.8 (C, C-8a), 122.6 (CH, CH_2_CCCHCH), 123.2 (CH, HNCH), 126.0 (C, C-4), 127.0 (C, 12-CH_2_CC), 135.7 (C, C-4a), 136.2 (C, HNC), 146.7 (C, C-7), 148.1 (CH, C-1), 162.8 (C, C-3), 168.8 (C, C-11), 170.8 (C, C-13), 179.8 (C, C-8), 180.6 (C, C-5); HRMS (M^+^): *m*/*z* calcd. for C_24_H_21_N_3_O_6_: 447.14304; found: 447.14845.

Compound **8b** (more polar, 29 mg, 0.06 mmol, 9%): orange solid, mp: 89–91 °C; IR ν_max_: 3370 (N-H), 2952 and 2925 (C-H), 1737 (C=O ester); 1686 (C=O quinone); ^1^H-NMR (400 MHz, CDCl_3_): δ 2.64 (s, 3H, 3-Me), 3.36 (dd, 1H, *J* = 6.4; 15 Hz, 12-CH_2_), 3.43 (dd, 1H, *J* = 5.2; 15 Hz, 12-CH_2_), 3.71 (s, 3H, 12-CO_2_Me), 4.01 (s, 3H, 4-CO_2_Me), 4.36 (m, 1H, 12-H), 5.66 (s, 1H, 7-H), 6.26 (d, 1H, *J* = 8.0 Hz, NH), 7.02 (m, 1H, CH), 7.12 (m, 1H, arom), 7.19 (m, 1H, arom), 7.35 (d, 1H, *J* = 8.0 Hz, arom), 7.50 (d, 1H, *J* = 8.0 Hz, arom), 8.23 (br s, 1H, NH), 9.24 (s, 1H, 1-H); ^13^C-NMR (100 MHz, CDCl_3_): δ 22.7 (CH_3_, C-9), 27.7 (CH_2_, 12-CH_2_), 53.0 (CH_3_, C-14), 53.4 (CH_3_, C-10), 55.5 (CH, C-12), 102.4 (CH, C-7), 109.1 (C, 12-CH_2_C), 111.6 (CH, HN-CCH), 118.4 (CH, 12-CH_2_CCCH), 120.1 (CH, HN-CCHCH), 122.7 (C and CH, C-8a y CH_2_CCCHCH), 123.1 (CH, HNCH), 125.0 (C, C-4), 127.1 (C, 12-CH_2_CC), 132.3 (C, C-4a), 136.3 (C, HNC), 146.4 (C, C-6), 148.7 (CH, C-1), 160.3 (C, C-3), 168.4 (C, C-11), 170.9 (C, C-13), 180.8 (C, C-5), 181.8 (C, C-8); HRMS (M^+^): *m*/*z* calcd. for C_24_H_21_N_3_O_6_: 447.14304; found: 447.14818.

*Methyl 6- and 7-(1-methoxy-1-oxopropan-2-ylamino)-3-methyl-5,8-dioxo-5,8-dihydroisoquinoline-4-carboxylate* (**9a**, **9b**). The mixture of regioisomers was prepared from **1** (200 mg, 0.87 mmol) and d-alanine methyl ester hydrochloride (2 h). Compound **9a** (less polar, 104 mg, 0.31 mmol, 50%): orange solid, mp: 140.5–142 °C; IR ν_max_ 3370 and 3324 (N-H), 2954 and 2930 (C-H), 1745 (C=O ester); 1687 (C=O quinone); ^1^H-NMR (400 MHz, CDCl_3_): δ 1.57 (d, 3H, *J* = 6.8 Hz, Me), 2.65 (s, 3H, 3-Me), 3.81 (s, 3H, 12-CO_2_Me), 4.03 (s, 3H, 4-CO_2_Me), 4.13 (m, 1H, 12-H), 5.70 (s, 1H, 6-H), 6.46 (d, 1H, *J* = 7.2 Hz, NH), 9.19 (s, 1H, 1-H); ^13^C-NMR (100 MHz, CDCl_3_): δ 17.6 (CH_3_, 12-CH_3_), 23.1 (CH_3_, C-9), 50.7 (CH, C-12), 53.1 (CH_3_, C-10), 53.2 (CH_3_, C-14), 102.4 (CH, C-6), 122.0 (C, C-8a), 126.2 (C, C-4), 135.8 (C, C-4a), 146.4 (C, C-7), 148.3 (CH, C-1), 163.0 (C, C-3), 168.8 (C, C-11), 171.7 (C, C-13), 180.1 (C, C-5), 180.7 (C, C-8); HRMS (M^+^): *m*/*z* calcd. for C_16_H_16_N_2_O_6_: 332.10084; found: 332.10193. Attempts to isolate a pure sample of **9b** were unsuccessful.

*Methyl 6- and 7-(1-methoxy-1-oxo-3-phenylpropan-2-ylamino)-3-methyl-5,8-dioxo-5,8-dihydroisoquinoline-4-carboxylate* (**10a**, **10b**). The mixture of regioisomers was prepared from **1** (200 mg, 0.87 mmol) and d-phenylalanine methyl ester hydrochloride (2 h 30 min).Compound **10a** (less polar, 139 mg, 0.34 mmol, 58%): orange solid, mp: 118.5–120 °C; IR ν_max_: 3353 and 3287 (N-H), 2947 and 2927 (C-H), 1735 (C=O ester); 1685 (C=O quinone); ^1^H-NMR (400 MHz, CDCl_3_): δ 2.64 (s, 3H, 3-Me), 3.17 (dd, 1H, *J* = 6.4; 14 Hz, 12-CH_2_), 3.26 (dd, 1H, *J* = 5.6; 14 Hz, 12-CH_2_), 3.76 (s, 3H, 12-CO_2_Me), 4.02 (s, 3H, 4-CO_2_Me), 4.31 (m, 1H, 12-H), 5.68 (s, 1H, 6-H), 6.42 (d, 1H, *J* = 7.6 Hz, NH), 7.11 (m, 2H, arom), 7.28 (m, 3H, arom), 9.16 (s, 1H, 1-H); ^13^C-NMR (100 MHz, CDCl_3_): δ 23.0 (CH_3_, C-9), 37.5 (CH_2_, 12-CH_2_), 52.9 (CH_3_, C-14), 53.2 (CH_3_, C-10), 56.1 (CH, C-12), 102.5 (CH, C-6), 121.8 (C, C-8a), 126.1 (C, C-4), 127.8 (C, C-4’), 129.0 (2CH, C2’ and C6’), 129.1 (2CH, C-3’ and C-5’), 134.8 (C, 12-CH_2_C1’), 135.6 (C, C-4a), 146.4 (C, C-7), 148.2 (CH, C-1), 163.0 (C, C-3), 168.7 (C, C-11), 170.3 (C, C-13), 179.9 (C, C-8), 180.6 (C, C-5); HRMS (M^+^): *m*/*z* calcd. for C_22_H_20_N_2_O_6_: 408.13214; found: 408.13759.

Compound **10b** (more polar, 12 mg, 0.03 mmol, 5%): orange solid, mp: 71.5–73 °C; IR ν_max_: 3350 and 3284 (N-H), 2946 and 2929 (C-H), 1735 (C=O ester); 1684 (C=O quinone); ^1^H-NMR (400 MHz, CDCl_3_): δ 2.65 (s, 3H, 3-Me), 3.14 (dd, 1H, *J* = 6.8; 14 Hz, 12-CH_2_), 3.25 (dd, 1H, *J* = 5.6; 14 Hz, 12-CH_2_), 3.76 (s, 3H, 12-CO_2_Me), 4.04 (s, 3H, 4-CO_2_Me), 4.31 (m, 1H, 12-H), 5.67 (s, 1H, 7-H), 6.18 (d, 1H, *J* = 7.6 Hz, NH), 7.13 (m, 2H, arom), 7.29 (m, 3H, arom), 9.26 (s, 1H, 1-H); ^13^C-NMR (100 MHz, CDCl_3_): δ 22.7 (CH_3_, C-9), 37.7 (CH_2_, 12-CH_2_), 53.0 (CH_3_, C-14), 53.4 (CH_3_, C-10), 56.3 (CH, C-12), 102.6 (CH, C-7), 122.7 (C, C-8a), 125.1 (C, C-4), 127.8 (C, C-4’), 129.1 (2CH, C-2’ and C-6’), 129.2 (2CH, C-3’ and C-5’), 132.3 (C, C-4a), 134.8 (C, 12-CH_2_C1’), 146.3 (C, C-6), 148.8 (CH, C-1), 160.4 (C, C-3), 168.4 (C, C-11), 170.5 (C, C-13), 180.8 (C, C-5), 181.8 (C, C-8); HRMS (M^+^): *m*/*z* calcd. for C_22_H_20_N_2_O_6_: 408.13214; found: 408.13757.

*Methyl 6- and 7-(3-(1H-indol-3-yl)-1-methoxy-1-oxopropan-2-ylamino)-3-methyl-5,8-dioxo-5,8-dihydroisoquinoline-4-carboxylate* (**11a**, **11b**). The mixture of regioisomers was prepared from **1** (200 mg, 0.87 mmol) and d-tryptophan methyl ester hydrochloride (2 h). Compound **11a** (less polar, 205 mg, 0.46 mmol, 66%): orange solid, mp: 99–100.5 °C; IR ν_max_: 3360 (N-H), 2952 and 2927 (C-H), 1737 (C=O ester); 1681 (C=O quinone); ^1^H-NMR (400 MHz, CDCl_3_): δ 2.63 (s, 3H, 3-Me), 3.35 (dd, 1H, *J* = 6.4; 15 Hz, 12-CH_2_), 3.40 (dd, 1H, *J* = 5.2; 15 Hz, 12-CH_2_), 3.70 (s, 3H, 12-Me), 4.01 (s, 3H, 4-CO_2_Me), 4.33 (m, 1H, 12-H), 5.64 (s, 1H, 6-H), 6.47 (d, 1H, *J* = 7.6 Hz, NH), 6.98 (m, 1H, CH), 7.07 (m, 1H, arom), 7.13 (m, 1H, arom), 7.28 (d, 1H, *J* = 8.0 Hz, arom), 7.45 (d, 1H, *J* = 8.0 Hz, arom), 8.45 (br s, 1H, NH), 9.10 (s, 1H, 1-H); ^13^C-NMR (100 MHz, CDCl_3_): δ 23.0 (CH_3_, C-9), 27.5 (CH_2_, 12-CH_2_), 53.0 (CH_3_, C-14), 53.2 (CH_3_, C-10), 55.5 (CH, C-12), 102.2 (CH, C-6), 108.8 (C, 12-CH_2_C), 111.6 (CH, HN-CCH), 118.2 (CH, 12-CH_2_CCCH), 120.0 (CH, HN-CCHCH), 121.8 (C, C-8a), 122.6 (CH, CH_2_CCCHCH), 123.2 (CH, HNCH), 126.0 (C, C-4), 127.0 (C, 12-CH_2_CC), 135.7 (C, C-4a), 136.2 (C, HNC), 146.7 (C, C-7), 148.1 (CH, C-1), 162.8 (C, C-3), 168.8 (C, C-11), 170.8 (C, C-13), 179.8 (C, C-8), 180.6 (C, C-5); HRMS (M^+^): *m*/*z* calcd. for C_24_H_21_N_3_O_6_: 447.14304; found: 447.14867. Attempts to isolate a pure sample of **11b** were unsuccessful.

#### 3.2.2. Synthesis of Bromoisoquinolinequinones **12**, **13a**,**b**, **14**, **15**, **16**. General Procedure

A solution of the required aminoisoquinolinequinone (1 equiv.), *N*-bromosuccinimide (NBS) (1.1 equiv.) and methanol (15 mL) was left with stirring at rt after completion of the reaction as indicated by TLC. The solvent was removed under reduced pressure and the residue was column chromatographed over silica gel (CH_2_Cl_2_/EtOAc 90:10) to yield the corresponding bromoisoquinolinequinones.

*Methyl 6-bromo-7-(1-methoxy-1-oxopropan-2-ylamino)-3-methyl-5,8-dioxo-5,8-dihydroisoquinoline-4-carboxylate* (**12**). Prepared from **2a** (70 mg, 0.21 mmol) and NBS (20 min, 65 mg, 0.16 mmol, 76% yield): orange solid, mp: 120.5–122 °C; IR ν_max_: 3370 and 3320 (N-H), 2954 and 2924 (C-H), 1738 (C=O ester); 1685 (C=O quinone); ^1^H-NMR (400 MHz, CDCl_3_): δ 1.62 (d, 3H, *J* = 6.8 Hz, 12-Me), 2.65 (s, 3H, 3-Me), 3.83 (s, 3H, 12-CO_2_Me), 4.05 (s, 3H, 4-CO_2_Me), 4.13 (m, 1H, 12-H), 6.55 (bs, 1H, NH), 9.17 (s, 1H, 1-H); ^13^C-NMR (100 MHz, CDCl_3_): δ 20.2 (CH_3_, 12-CH_3_), 21.1 (CH_3_, C-9), 52.6 (CH, C-12), 53.1 (CH_3_, C-10), 53.3 (CH_3_, C-14), 121.3 (C, C-8a), 126.3 (C, C-4), 134.1 (C, C-4a), 148.7 (2C, C, C-7 and CH, C-1), 163.1 (C, C-3), 168.2 (C, C-11), 172.7 (C, C-13), 178.5 (2C, C-5 and C-8); HRMS (M^+^): *m*/*z* calcd. for C_16_H_15_BrN_2_O_6_: 410.01135; found: 410.01755.

*Methyl 6-bromo-(1-methoxy-3-methyl-1-oxobutan-2-ylamino)-3-methyl-5,8-dioxo-5,8-dihydroisoquinoline-4-carboxylate* (**13a**). Prepared from **3a** (100 mg, 0.28 mmol) and NBS (20 min, 101 mg, 0.23 mmol, 82% yield): orange solid, mp: 105.2–107 °C; IR ν_max_: 3368 (N-H), 2965 and 2934 (C-H), 1725 (C=O ester); 1680 (C=O quinone); ^1^H-NMR (400 MHz, CDCl_3_): δ 1.02 (d, 3H, *J* = 6.8 Hz, 12-C-Me), 1.08 (d, 3H, *J* = 6.8 Hz, 12-C-Me), 2.33 (m, 1H, 12-CH), 2.65 (s, 3H, 3-Me), 4.03 (s, 3H, 12-CO_2_Me), 4.05 (s, 3H, 4-CO_2_Me), 4.13 (m, 1H, 12-H), 6.22 (bs, 1H, NH), 9.18 (s, 1H, 1-H); ^13^C-NMR (100 MHz, CDCl_3_): δ 17.9 (CH_3_, 12-CHCH_3_), 18.4 (CH_3_, 12-CHCH_3_), 23.1 (CH_3_, C-9), 32.5 (CH, 12-CH), 52.7 (CH_3_, C-14), 53.3 (CH_3_, C-10), 60.5 (CH, C-12), 121.4 (C, C-8a), 126.4 (C, C-4), 135.6 (C, C-4a), 148.7 (2C, C-7 and CH, C-1), 163.1 (C, C-3), 168.2 (C, C-11), 171.2 (C, C-13), 178.6 (2C, C-5 and C-8); HRMS (M^+^): *m*/*z* calcd. for C_18_H_19_N_2_O_6_Br: 438.04265; found: 438.04810.

*Methyl 7-bromo-(1-methoxy-3-methyl-1-oxobutan-2-ylamino)-3-methyl-5,8-dioxo-5,8-dihydroisoquinoline-4-carboxylate* (**13b**). Prepared from **3b** (50 mg, 0.14 mmol) and NBS (30 min, 49 mg, 0.11 mmol, 78% yield): orange solid, mp: 105–106.6 °C; IR ν_max_: 3370 (N-H), 2962 and 2931 (C-H), 1743 (C=O ester); 1682 (C=O quinone); ^1^H-NMR (400 MHz, CDCl_3_): δ 1.01 (d, 3H, *J* = 6.8 Hz, 12-C-Me), 1.07 (d, 3H, *J* = 6.8 Hz, 12-C-Me), 2.29 (m, 1H, 12-C-CH), 2.65 (s, 3H, 3-Me), 3.80 (s, 3H, 12-CO_2_Me), 4.04 (m, 1H, 12-H), 4.05 (s, 3H, 4-CO_2_Me), 6.30 (bs, 1H, NH), 9.29 (s, 1H, 1-H); ^13^C-NMR (100 MHz, CDCl_3_): δ 17.8 (CH_3_, 12-CHCH_3_), 18.5 (CH_3_, 12-CHCH_3_), 22.8 (CH_3_, C-9), 32.5 (CH, 12-CH), 52.7 (CH_3_, C-14), 53.3 (CH_3_, C-10), 61.6 (CH, C-12), 121.8 (C, C-8a), 125.4 (2C, C-4 and C-4a), 146.9 (C, C-6), 149.0 (2C, CH, C-1 and C, C-3), 168.0 (C, C-11), 171.5 (C, C-13), 179.3 (2C, C-5 and C-8); HRMS (M^+^): *m*/*z* calcd. for C_18_H_19_N_2_O_6_Br: 438.04265; found: 438.04785.

*Methyl 6-bromo-(1-methoxy-4-methyl-1-oxopentan-2-ylamino)-3-methyl-5,8-dioxo-5,8-dihydroisoquinoline-4-carboxylate* (**14**). Prepared from **4a** (100 mg, 0.27 mmol) and NBS (30 min, 98 mg, 0.22 mmol, 80% yield): orange solid, mp: 104.3–106.2 °C; IR ν_max_: 3370 and 3326 (N-H), 2945 and 2914 (C-H), 1755 (C=O ester); 1710 (C=O quinone); ^1^H-NMR (400 MHz, CDCl_3_): δ 0.98 (d, 3H, *J* = 6.4 Hz, 12-CH_2_-C-Me), 1.01 (d, 3H, *J* = 6.4 Hz, 12-CH_2_-C-Me), 1.81 (m, 3H, 12-CH_2_-CH and 12-CH_2_), 2.65 (s, 3H, 3-Me), 3.79 (s, 3H, 12-CO_2_Me), 4.07 (s, 4H, 4-CO_2_Me and 12-H), 6.21 (bs, 1H, NH), 9.17 (s, 1H, 1-H); ^13^C-NMR (100 MHz, CDCl_3_): δ 22.5 (CH_3_, 2-CHCH_3_), 22.8 (CH_3_, 12-CHCH_3_), 23.0 (CH_3_, C-9), 25.1 (CH, 12-CH_2_CH), 42.6 (CH, 12-CH), 52.8 (CH_3_, C-14), 53.3 (CH_3_, C-10), 53.5 (CH, C-12), 121.3 (C, C-8a), 126.4 (C, C-4), 134.1 (C, C-4a), 148.7 (2C, C-7 and CH, C-1), 163.0 (C, C-3), 168.1 (C, C-11), 171.1 (C, C-13), 178.5 (2C, C-5 and C-8); HRMS (M^+^): *m*/*z* calcd. for C_19_H_21_N_2_O_6_Br: 452.05830; found: 452.06328.

*Methyl 6-bromo-(1-methoxy-1-oxo-3-phenylpropan-2-ylamino)-3-methyl-5,8-dioxo-5,8-dihydroisoquinoline-4-carboxylate* (**15**). Prepared from **6a** (100 mg, 0.25 mmol) and NBS (40 min, 83 mg, 0.17 mmol, 68% yield): red solid, mp: 125.2–127.5 °C; IR ν_max_: 3350 and 3285 (N-H), 2944 and 2921 (C-H), 1746 (C=O ester); 1705 (C=O quinone); ^1^H-NMR (400 MHz, CDCl_3_): δ 2.63 (s, 3H, 3-Me), 3.25 (dd, 1H, *J* = 6.4; 14 Hz, 12-CH_2_), 3.27 (dd, 1H, *J* = 5.6; 14 Hz, 12-CH_2_), 3.79 (s, 3H, 12-CO_2_Me), 4.03 (s, 4H, 4-CO_2_Me and 12-H), 6.31 (bs, 1H, NH), 7.13 (m, 2H, arom), 7.28 (m, 3H, arom), 9.09 (s, 1H, 1-H); ^13^C-NMR (100 MHz, CDCl_3_): δ 22.9 (CH_3_, C-9), 39.9 (CH_2_, 12-CH_2_), 52.9 (CH_3_, C-14), 53.3 (CH_3_, C-10), 57.7 (CH, C-12), 121.5 (C, C-8a), 126.1 (C, C-4), 127.8 (C, C-4’), 129.1 (2CH, C-2’ and C-6’), 129.4 (2CH, C-3’ and C-5’), 134.6 (2C, 12-CH_2_C1’ and C, C-4a), 148.6 (2C, C-7 and CH, C-1), 163.0 (C, C-3), 168.2 (C, C-11), 171.3 (C, C-13), 178.5 (2C, C-5 and C-8); HRMS (M^+^): *m*/*z* calcd. for C_22_H_19_N_2_O_6_Br: 486.04265; found: 486.04732.

*Methyl 6-bromo-(1-methoxy-1-oxo-3-phenylpropan-2-ylamino)-3-methyl-5,8-dioxo-5,8-dihydroisoquinoline-4-carboxylate* (**16**). Prepared from **10a** (80 mg, 0.19 mmol) and NBS (40 min, 63 mg, 0.13 mmol, 68% yield) red solid, mp: 126–127 °C; IR ν_max_: 3352 and 3285 (N-H), 2950 and 2927 (C-H), 1725 (C=O ester); 1715 (C=O quinone); ^1^H-NMR (400 MHz, CDCl_3_): δ 2.63 (s, 3H, 3-Me), 3.19 (dd, 1H, *J* = 6.4; 14 Hz, 12-CH_2_), 3.26 (dd, 1H, *J* = 5.6; 14 Hz, 12-CH_2_), 3.79 (s, 3H, 12-CO_2_Me), 4.05 (s, 4H, 4-CO_2_Me and 12-H), 6.29 (bs, 1H, NH), 7.13 (m, 2H, arom), 7.25 (m, 3H, arom), 9.08 (s, 1H, 1-H); ^13^C-NMR (100 MHz, CDCl_3_): 22.9 (CH_3_, C-9), 39.8 (CH_2_, 12-CH_2_), 52.9 (CH_3_, C-14), 53.2 (CH_3_, C-10), 57.7 (CH, C-12), 121.4 (C, C-8a), 126.1 (C, C-4), 127.7 (C, C-4’), 128.9 (2CH, C-2’ and C-6’), 129.4 (2CH, C-3’ and C-5’), 134.6 (2C, 12-CH_2_C1’ and C, C-4a), 148.5 (2C, C-7 and CH, C-1), 163.0 (C, C-3), 168.1 (C, C-11), 171.3 (C, C-13), 178.4 (2C, C-5 and C-8); HRMS (M^+^): *m*/*z* calcd. for C_22_H_19_N_2_O_6_Br: 486.04265; found: 486.04723.

### 3.3. In Vitro Cytotoxic Evaluation

*Cytotoxicity assay (Table 3 and Table 4)*. Cell Lines and Culture Conditions: MRC-5 normal human lung fibroblasts (CCL-171), AGS human gastric adenocarcinoma cells (CRL-1739), SK-MES-1 human lung cancer cells (HTB-58), and J82 human bladder carcinoma cells (HTB-1) were obtained from the American Type Culture Collection (ATCC, Manassas, VA, USA). MRC-5, SK-MES-1, and J82 cells were grown in Eagle’s minimal essential medium (EMEM) containing 2 mM l-glutamine, 1 mM sodium pyruvate and 1.5 g/L sodium hydrogen carbonate. AGS cells were grown in Ham F-12 supplemented with 2 mM l-glutamine and 1.5 g/L sodium hydrogen carbonate. Finally, HL-60 cells were grown in RPMI medium. Media were supplemented with 10% heat-inactivated fetal bovine serum (FBS), 100 IU/mL penicillin and 100 μg/mL streptomycin and cell cultures were kept in a humidified incubator with 5% CO_2_ in air at 37 °C. For the cytotoxicity experiments, cells were seeded into 96-well plates at a density of 50,000 cells/mL. After reaching confluence, cells were incubated for three days with compounds at varied concentrations ranging from 0 up to 100 μM while untreated cells (medium containing 1% DMSO) were used as controls. Cytotoxicity was assessed using the MTT (3-(4,5-dimethylthiazol-2-yl)-2,5-diphenyltetrazolium bromide) reduction assay. MTT was used at 1 mg/mL and the blue formazan crystals, formed during MTT reduction, were dissolved adding 100 μL of ethanol (acidified with HCl). The absorbance was measured at 550 nm using a Universal Microplate Reader (ELX 800, Bio-Tek Instruments Inc., Winooski, VT, USA). Values were the means of six replicates for each concentration and transformed to percentage of controls. The IC_50_ value was graphically obtained from the dose–response curves by adjusting them to a sigmoidal model (a + (b − a)/1 + 10(x − c)), where c = log IC_50_.

*Cytotoxicity assay (Table 5).* Cell Lines and Culture Conditions: Human cancer cells (T24, DU-145, MCF-7) and normal human fibroblasts (AG 1523 cells) were purchased from ATCC (Manassas, VA, USA) and cultured in high-glucose Dulbecco’s modified Eagle medium (Gibco, Grand Island, NY, USA) supplemented with 10% fetal calf serum, penicillin (100 U/mL), and streptomycin (100 μg/mL). All cultures were maintained in a humidified incubator at 37 °C under 5% CO_2_. Cells were incubated as monolayer at 37 °C for 48 h with DMSO (control conditions) and varied doses of compound **14**, tamoxifen, and 5-fluorouracil. The cytotoxic effect of compounds on cell lines was assessed using the MTT assay as reported previously [24]. Briefly, 10,000 cells/well were plated for 24 h in 96-well plates and, after reaching confluence, the cells were incubated for 48 h with DMSO or in the presence of compounds. Cells were washed twice with warm phosphate-buffered saline (PBS) and further incubated for 2 h with MTT (0.5 mg/mL). The blue formazan crystals were then solubilized by adding 100 μL DMSO/well and optical density of colored solutions was read at 550 nm. Results are expressed as % of MTT reduction compared to untreated control conditions. The calculation of IC_50_ values was performed by using GraphPad Prism software (San Diego, CA, USA).

## 4. Conclusions

In conclusion, we have synthesized a series of isoquinolinequinone-amino acid derivatives from 3-methyl-4-methoxycarbonylisoquinoline-5,8-quinone **1** and diverse l- and d-α-amino acid methyl esters. The cytotoxic activity of these isoquinolinequinone-amino acid derivatives and a number of bromine derivatives was evaluated by using the MTT assay. From the current investigation, structure-activity relationships of the aminoisoquinolinequinones demonstrate that compounds **2a**, **6a**, **10a**, and **14**, derived from l-alanine, l-phenylalanine, d-phenylalanine, and l-leucine, respectively, are endowed with high cytotoxic potencies and selectivity against cancer cells (gastric, bladder, lung, prostate, and breast). Also, it was deduced that the structure of the amino acid fragment plays a role on the cytotoxic effects and selectivity index. The major incidence of this structural effect is observed in the enantiomeric pair *S*-**2a**/*R*-**9a**, derived from alanine. Among the tested compounds in this study, the bromine derivative **14**, containing the l-leucine moiety at the 7-position, was found to be the most promising congener in the series, on account of its potency and selectivity to inhibit the growth of cancer cells. Compound **14** of novel structure, possessing significant cytotoxic activity on cancer cells, with relative fairly high MSI values, appears to be a new lead compound in the aminoisoquinolinequinone series.

## Figures and Tables

**Table 1:**
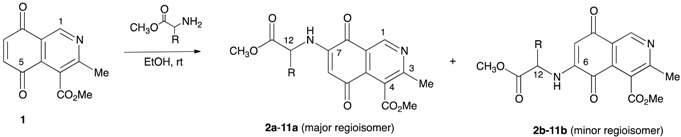
Reaction of isoquinolinequinone **1** with α-amino acid methyl esters.

α-Amino Acid	R	Products	a/b ^a^	Yield a/b (%) ^b^
l-ala	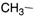	**2a** + **2b**	84/16	53/- ^c^
l-val	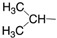	**3a** + **3b**	88/12	58/7
l-leu	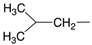	**4a** + **4b**	87/13	56/7
l-met	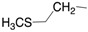	**5a** + **5b**	89/11	52/- ^c^
l-phe	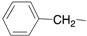	**6a** + **6b**	89/11	60/7
l-tyr	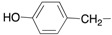	**7a** + **7b**	90/10	56/5
l-trp	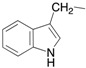	**8a** + **8b**	86/14	67/9
d-ala	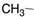	**9a** + **9b**	85/15	50/- ^c^
d-phe	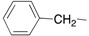	**10a** + **10b**	90/10	58/5
d-trp	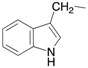	**11a** + **11b**	85/15	66/- ^c^

^a^ Determined by ^1^H-NMR analysis; ^b^ Isolated yields; ^c^ Not isolated.

**Table 2:**
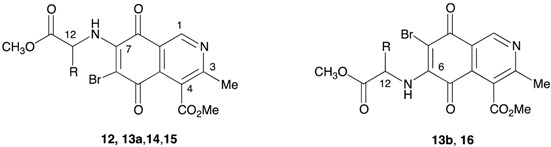
Yields of the synthesized bromine derivatives **12**, **13a**, **13b**, **14**, **15**, and **16**.

R	Precursor	Product	Yield (%) ^a^	R	Precursor	Product	Yield (%) ^a^
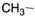	**2a**	**12**	78	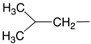	**4a**	**14**	69
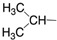	**3a**	**13a**	82	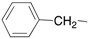	**6a**	**15**	76
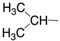	**3b**	**13b**	85	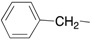	**10a**	**16**	64

^a^ Isolated yields.

**Table 3:** In vitro cytotoxic effect of compounds **2a**–**11a** and **3b**, **4b**, **6b**–**8b**, and **10b** on human-derived tumor cell lines: AGS (gastric), SK-MES-1 (lung), J82 (bladder), and the non-tumor fibroblasts (MRC-5).

No.	IC_50_ ± SEM ^a^ (μM)				
	MRC-5 ^b^	AGS ^c^	SK-MES-1 ^d^	J82 ^e^	MSI ^f^
**2a**	9.04 ± 0.69	2.25 ± 0.15	2.65 ± 0.19	6.25 ± 0.44	2.43
**3a**	4.94 ± 0.25	1.94 ± 0.08	4.62 ± 0.28	7.49 ± 0.44	1.10
**3b**	1.56 ± 0.09	0.69 ± 0.03	1.43 ± 0.09	2.19 ± 0.09	1.10
**4a**	5.70 ± 0.29	2.97 ± 0.15	4.56 ± 0.31	7.65 ± 0.51	1.13
**4b**	1.77 ± 0.14	1.59 ± 0.07	2.22 ± 0.12	2.35 ± 0.09	0.86
**5a**	5.22 ± 0.31	2.54 ± 0.21	2.93 ± 0.11	6.00 ± 0.32	1.42
**6a**	6.73 ± 0.34	1.85 ± 0.13	2.50 ± 0.15	4.68 ± 0.23	2.24
**6b**	2.16 ± 0.13	1.97 ± 0.12	1.91 ± 0.11	4.38 ± 0.28	0.79
**7a**	9.94 ± 0.51	2.61 ± 0.18	5.14 ± 0.35	9.67 ± 0.58	1.71
**7b**	3.74 ± 0.26	1.53 ± 0.06	2.57 ± 0.12	7.11 ± 0.55	1.00
**8a**	6.62 ± 0.46	2.54 ± 0.18	3.63 ± 0.18	7.56 ± 0.52	1.45
**8b**	1.72 ± 0.07	0.58 ± 0.03	1.34 ± 0.05	3.18 ± 0.19	0.91
**9a**	19.45 ± 1.36	4.75 ± 0.39	8.5 ± 0.59	15.43 ± 1.05	2.02
**10a**	7.46 ± 0.61	1.27 ± 0.11	2.29 ± 0.18	4.93 ± 0.29	2.63
**10b**	1.78 ± 0.12	1.98 ± 0.11	1.20 ± 0.05	3.03 ± 0.17	0.82
**11a**	4.99 ± 0.34	1.28 ± 0.09	2.58 ± 0.21	6.44 ± 0.51	1.45
**Etoposide**	0.33 ± 0.02	0.58 ± 0.02	1.83 ± 0.09	3.49 ± 0.16	0.16

^a^ Data represent average values of six independent determinations, SEM: standard error of the mean; ^b^ Normal human lung fibroblasts; ^c^ Human gastric adenocarcinoma cell line; ^d^ Human lung cancer cell line; ^e^ Human bladder carcinoma cell line; ^f^ MSI: Mean Selective Index = IC_50_ values for fibroblasts/IC_50_ values tumor cells.

**Table 4:** In vitro cytotoxic effect of the bromine compounds **12**, **13a**, **13b**, **14**, **15**, and **16** on human-derived tumor cell lines: AGS (gastric), SK-MES-1 (lung), J82 (bladder), and the non-tumor fibroblasts (MRC-5).

N°	IC_50_ ± SEM (μM)				
	MRC-5	AGS	SK-MES-1	J82	MSI
**12**	2.86 ± 0.17	2.07 ± 0.11	1.96 ± 0.11	3.76 ± 0.26	1.10
**13a**	2.98 ± 0.21	1.92 ± 0.13	2.28 ± 0.09	4.01 ± 0.19	1.15
**13b**	2.11 ± 0.11	2.70 ± 0.14	3.90 ± 0.31	8.76 ± 0.61	0.41
**14**	>100	>100	0.92 ± 0.04	2.21 ± 0.17	- ^a^
**15**	4.48 ± 0.27	2.08 ± 0.13	2.13 ± 0.12	4.61 ± 0.36	1.52
**16**	3.26 ± 0.13	2.19 ± 0.09	1.67 ± 0.12	6.08 ± 0.42	0.98
**Etoposide**	0.33 ± 0.02	0.58 ± 0.02	1.83 ± 0.09	3.49 ± 0.16	0.16

^a^ This value was not calculated because the IC_50_ values obtained for gastric adenocarcinoma cells and fibroblasts were higher than 100 μM.

**Table 5:** In vitro cytotoxic effect of compound **14** on human-derived tumor cell lines: T24 (bladder), DU-145 (prostate), MCF7 (breast) and healthy non-tumor fibroblasts (AG 1523).

Compound	IC_50_ ± SEM ^a^ (μM)				
	AG 1523 ^b^	T24 ^c^	DU-145 ^d^	MCF-7 ^e^	SI
**14**	1.57 ± 0.20	0.67 ± 0.06	0.64 ± 0.06	0.50 ± 0.05	2.62
Tamoxifen	22.5 ± 2.90	27.9 ± 3.50	22.9 ± 3.40	19.6 ± 0.85	0.85
5-Fluorouracil	6.6 ± 1.10	3.6 ± 0.25	7.1 ± 0.85	12.7 ±1.91	0.96

^a^ Data represent mean average values ± SEM of three separate experiments; ^b^ AG 1523 Healthy non-tumor fibroblasts; ^c^ T24 (bladder); ^d^ DU-145 (prostate); ^e^ MCF-7 (breast).

## Supplementary Materials

The following are available online at: http://www.mdpi.com/1420-3049/21/9/1199/s1, Figures S1–S7: ^1^H- and ^13^C-NMR of compounds **2a**, **3a**, **3b**, **11a**, **11b**, **13b**, and **15**.
