# Supplementary material for: Synthesis and Cytotoxic Activity on Human Cancer Cells of Novel Isoquinolinequinone–Amino Acid Derivatives
Source: Molecules. 2016 Sep 8;21(9):1199. doi: 10.3390/molecules21091199 (PMC6274474; doi:10.3390/molecules21091199)
Supplement: Supplementary file 1 [file molecules-21-01199-s001.pdf]

Supplementary Material: Synthesis and Cytotoxic Activity on Human Cancer Cells of Novel Isoquinolinequinone-Amino Acid Derivatives

Jaime A. Valderrama, Virginia Delgado, Sandra Sepúlveda, Julio Benites, Cristina Theoduloz, Pedro Buc Calderon, and Giulio G. Muccioli

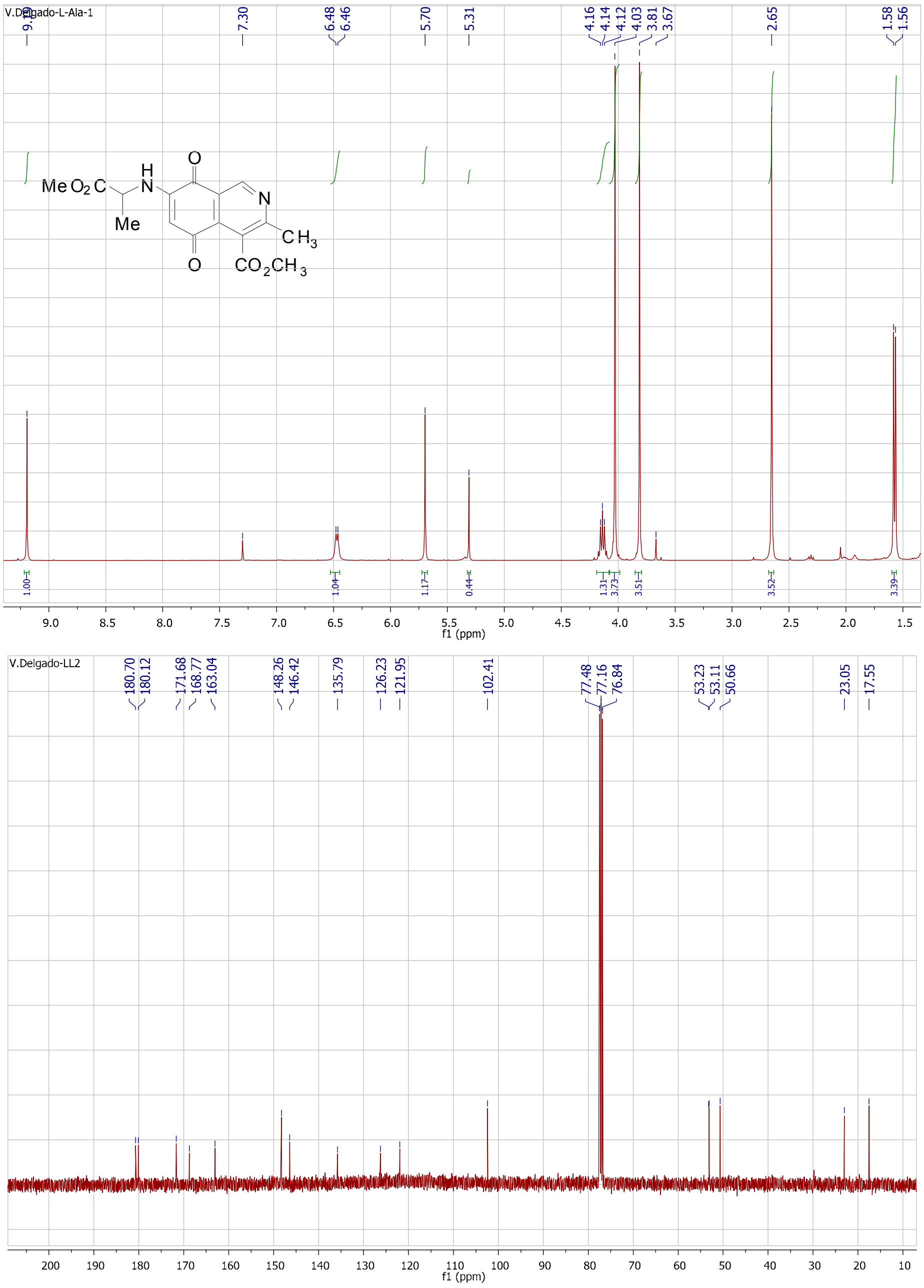

Figure S1. ^{1}H- and ^{13}C-NMR of compound **2a**.

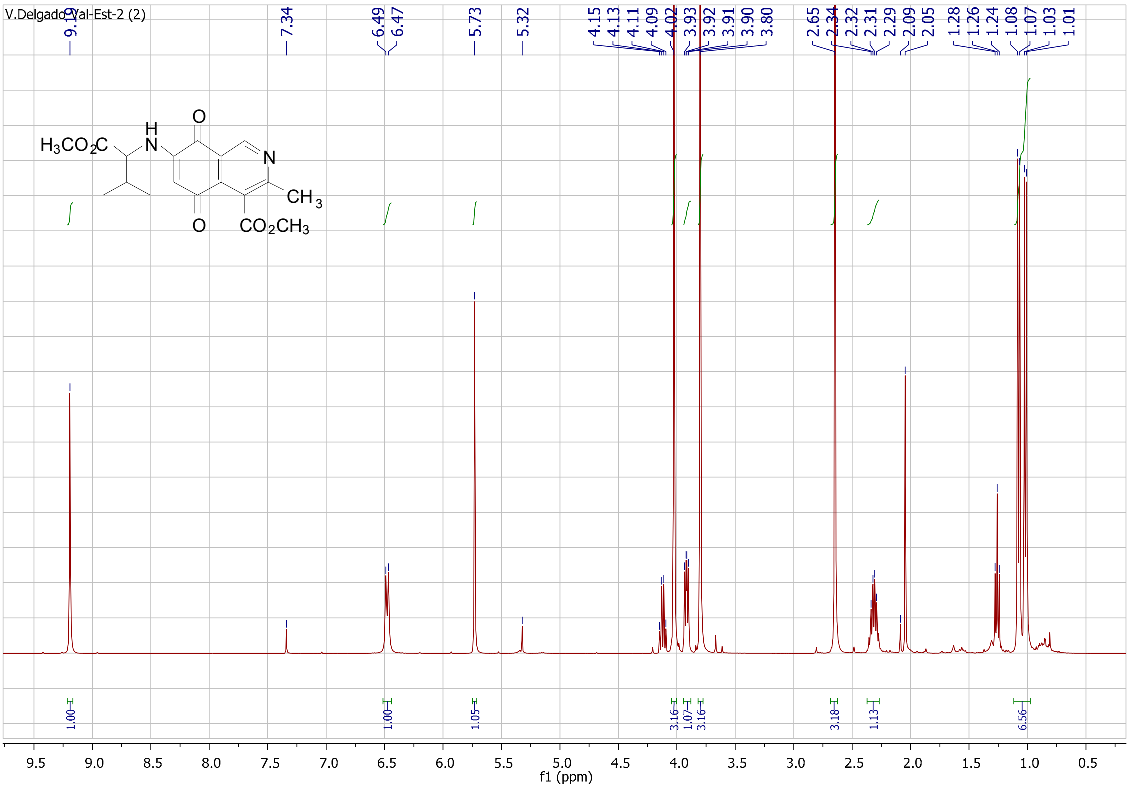

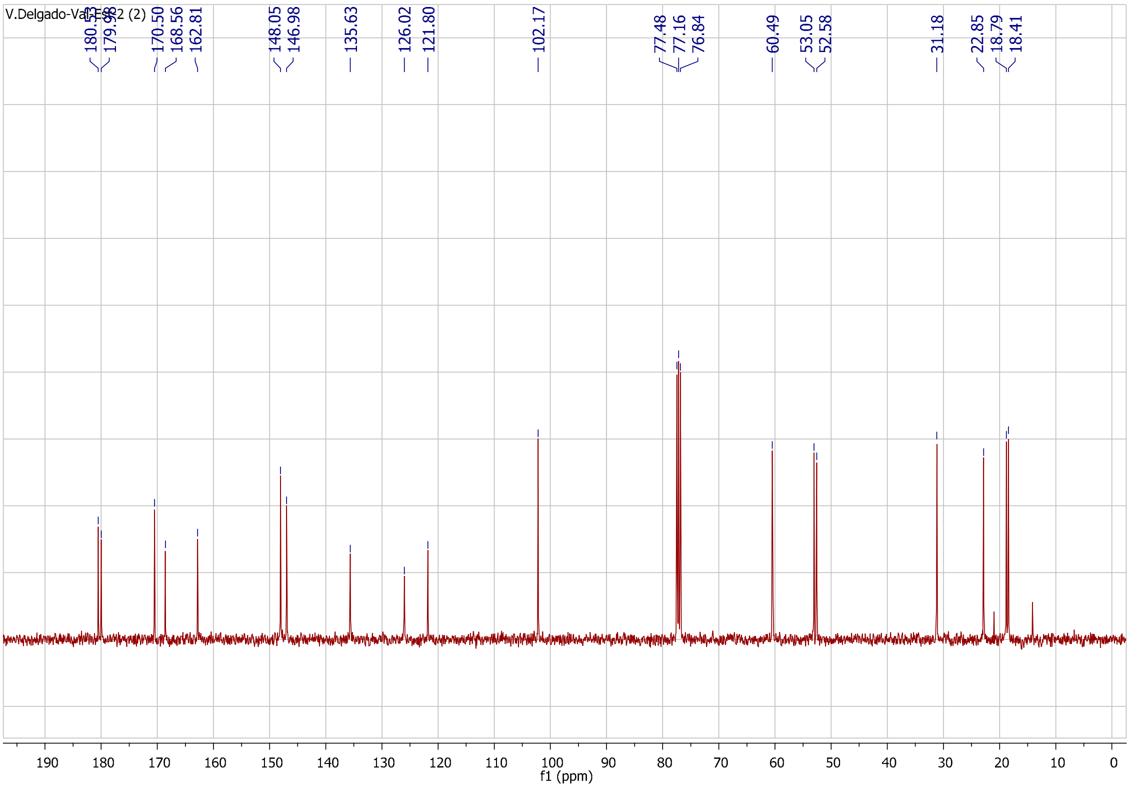

Figure S2. ^{1}H- and ^{13}C-NMR of compound **3a**.

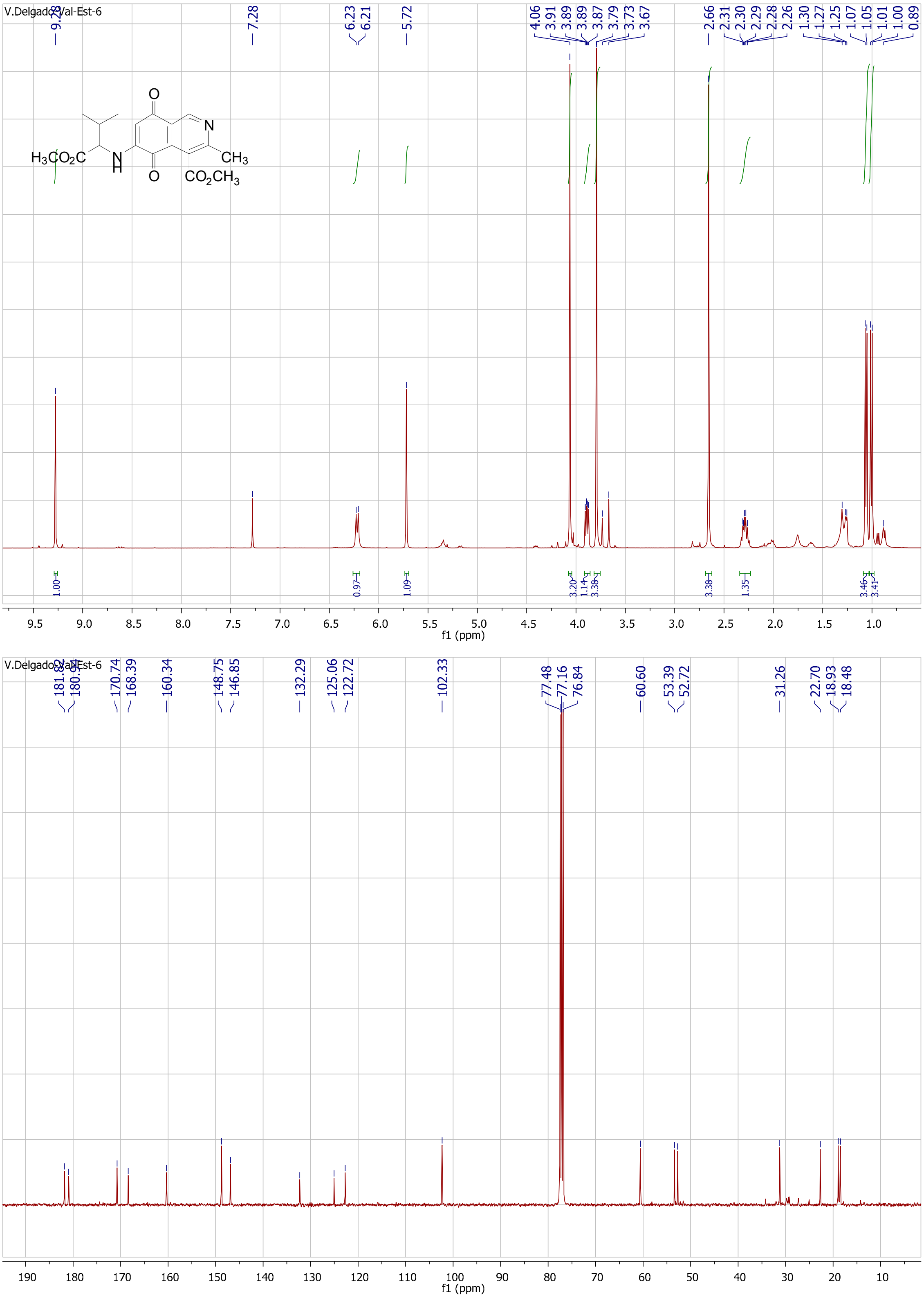

Figure S3. ¹H- and ¹³C-NMR of compound **3b**.

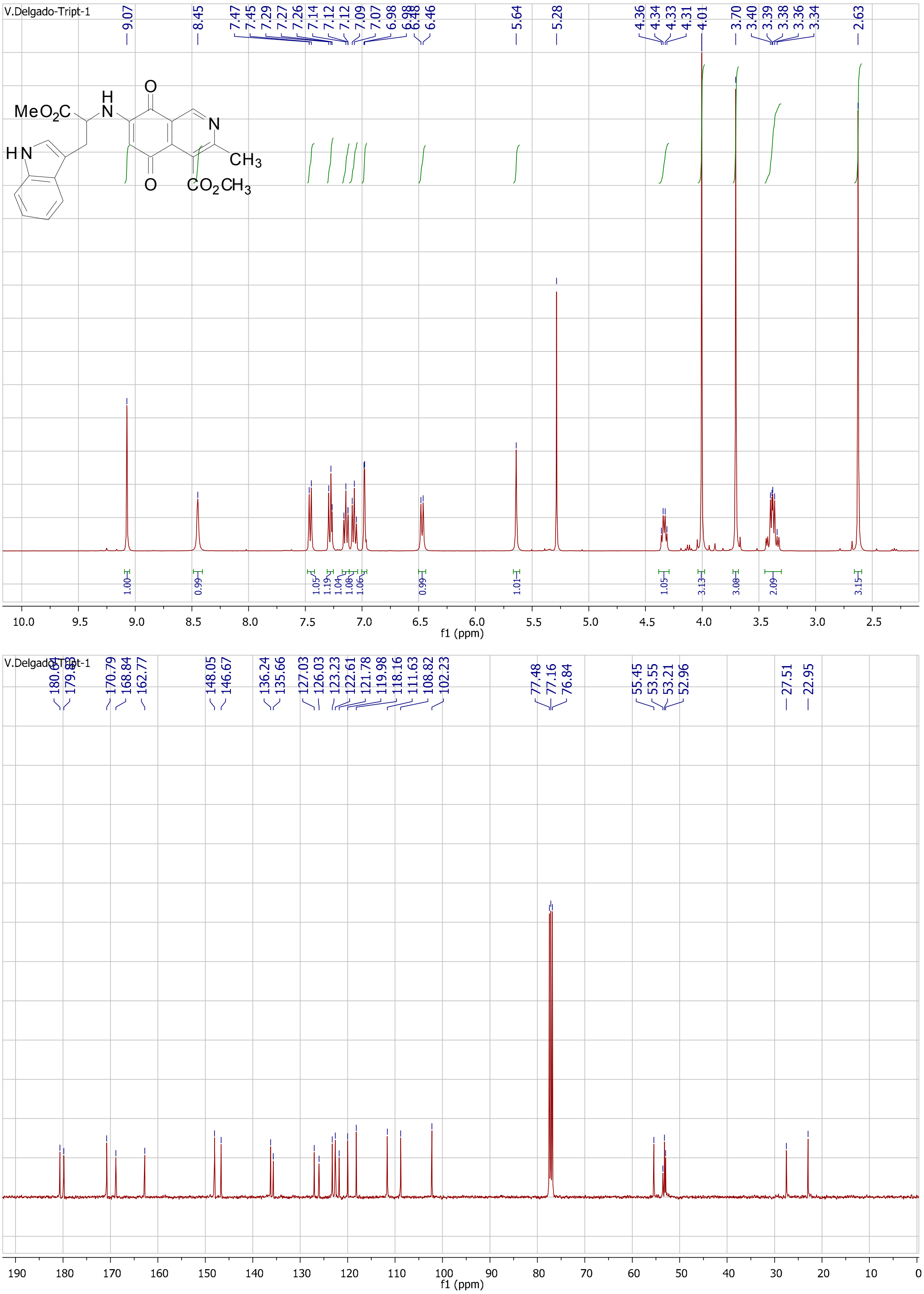

Figure S4. ^{1}H- and ^{13}C-NMR of compound **11a**.

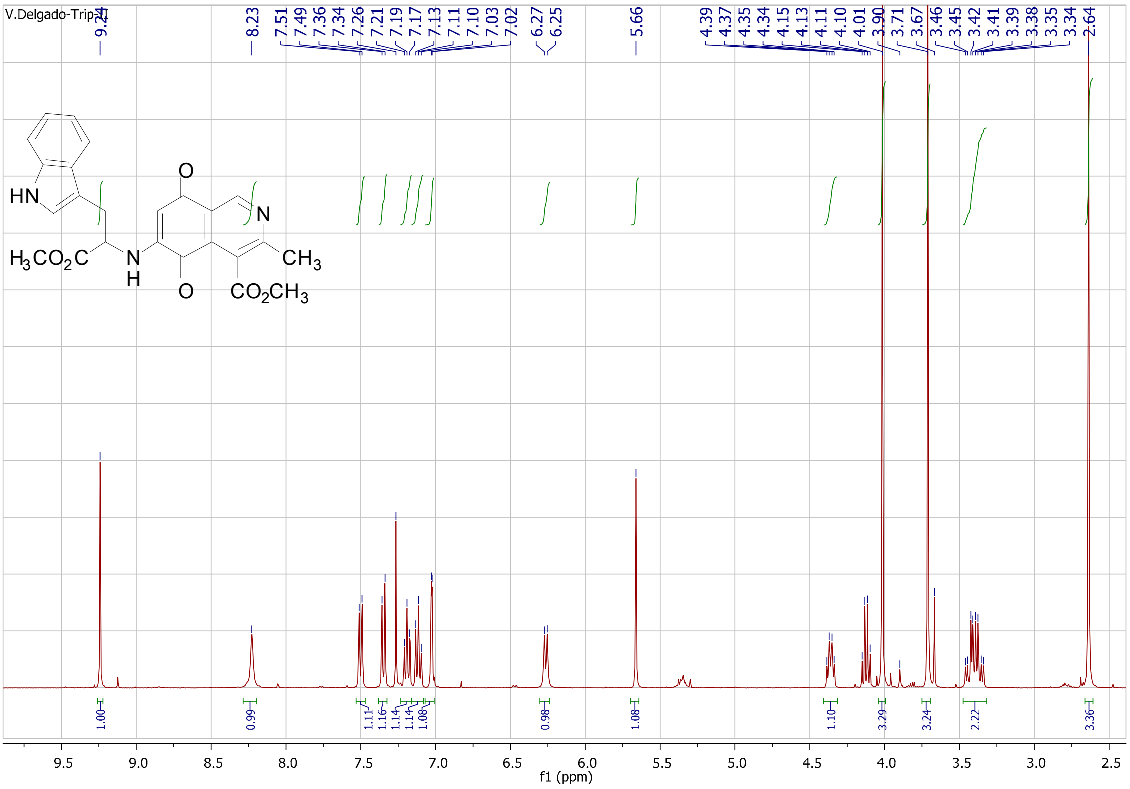

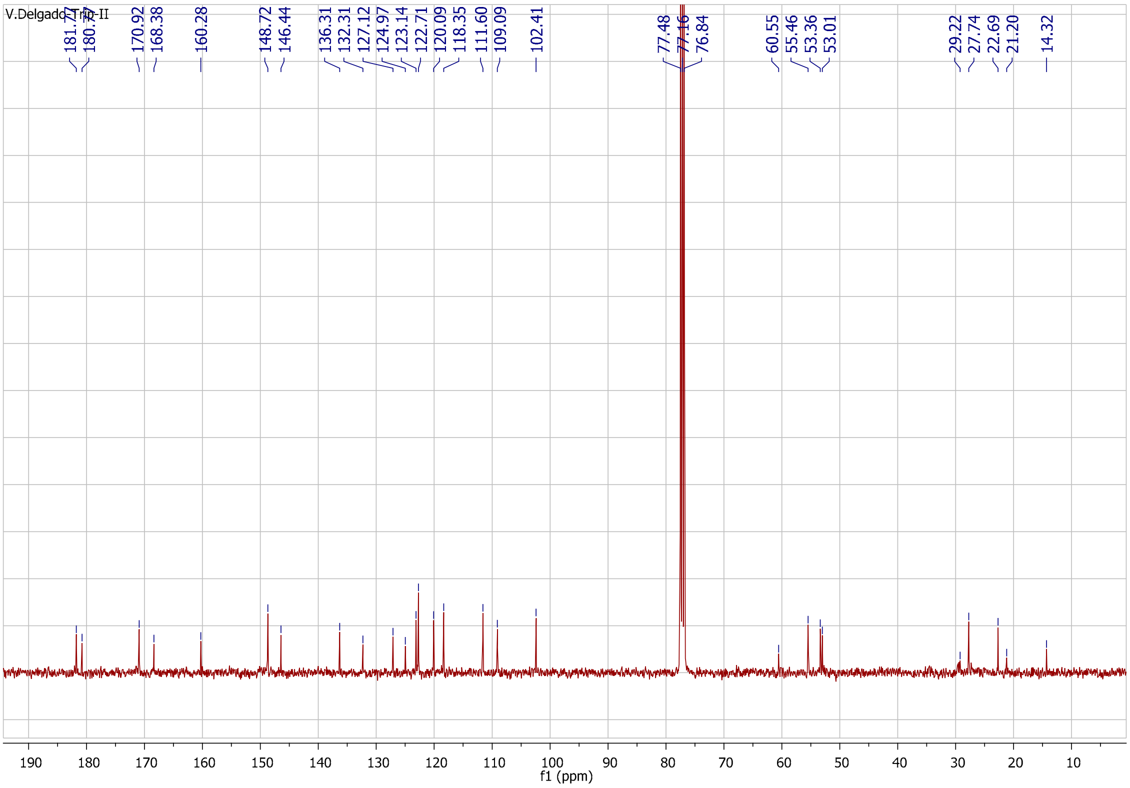

Figure S5. ^{1}H- and ^{13}C-NMR of compound **11b**.

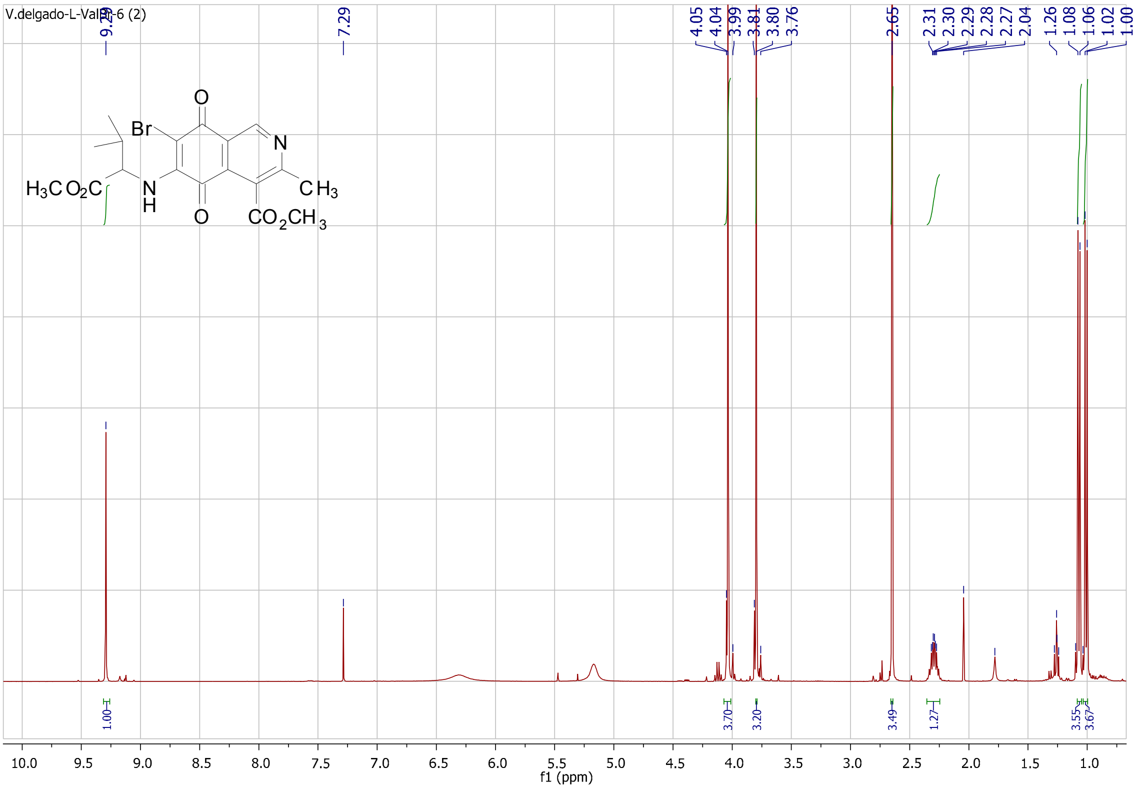

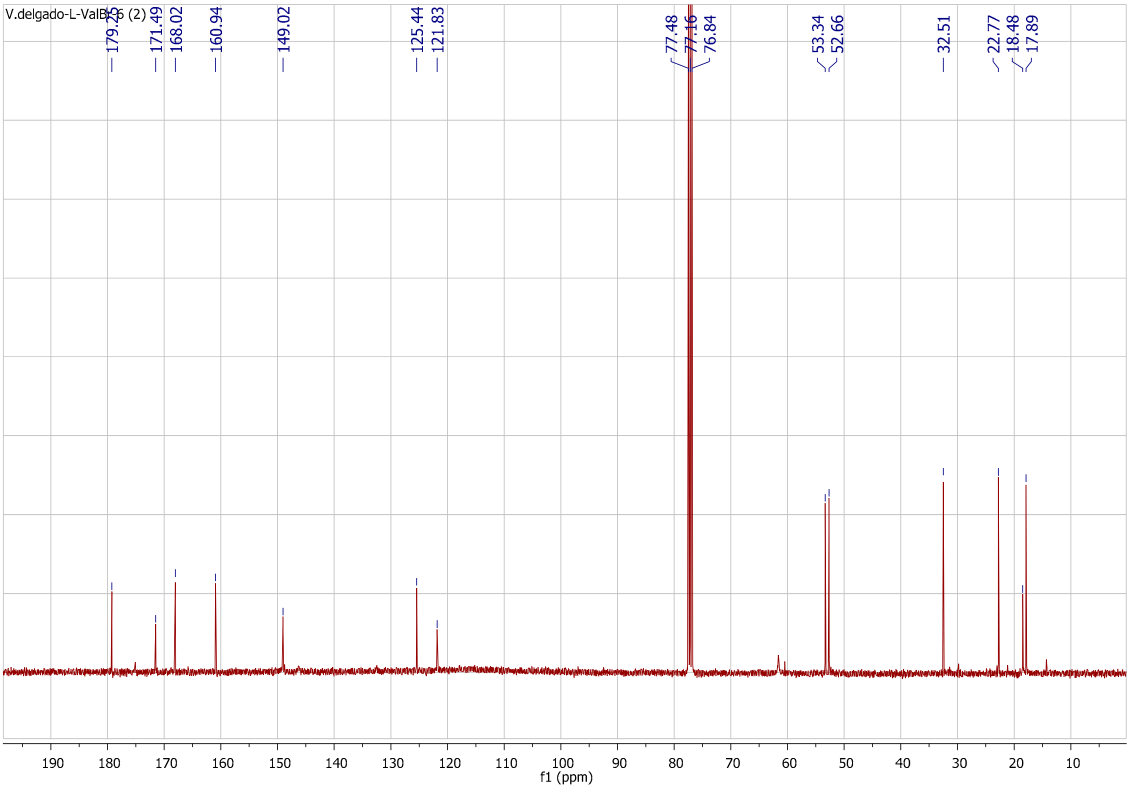

Figure S6. ^{1}H- and ^{13}C-NMR of compound **13b**.

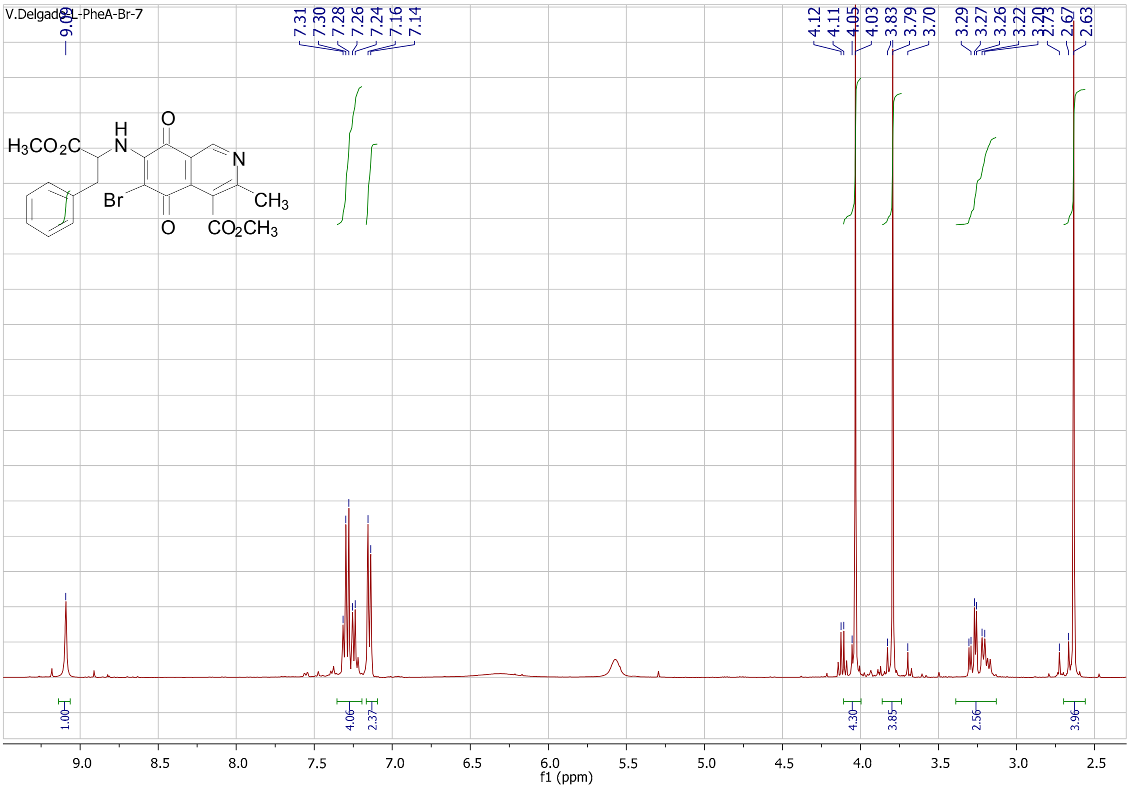

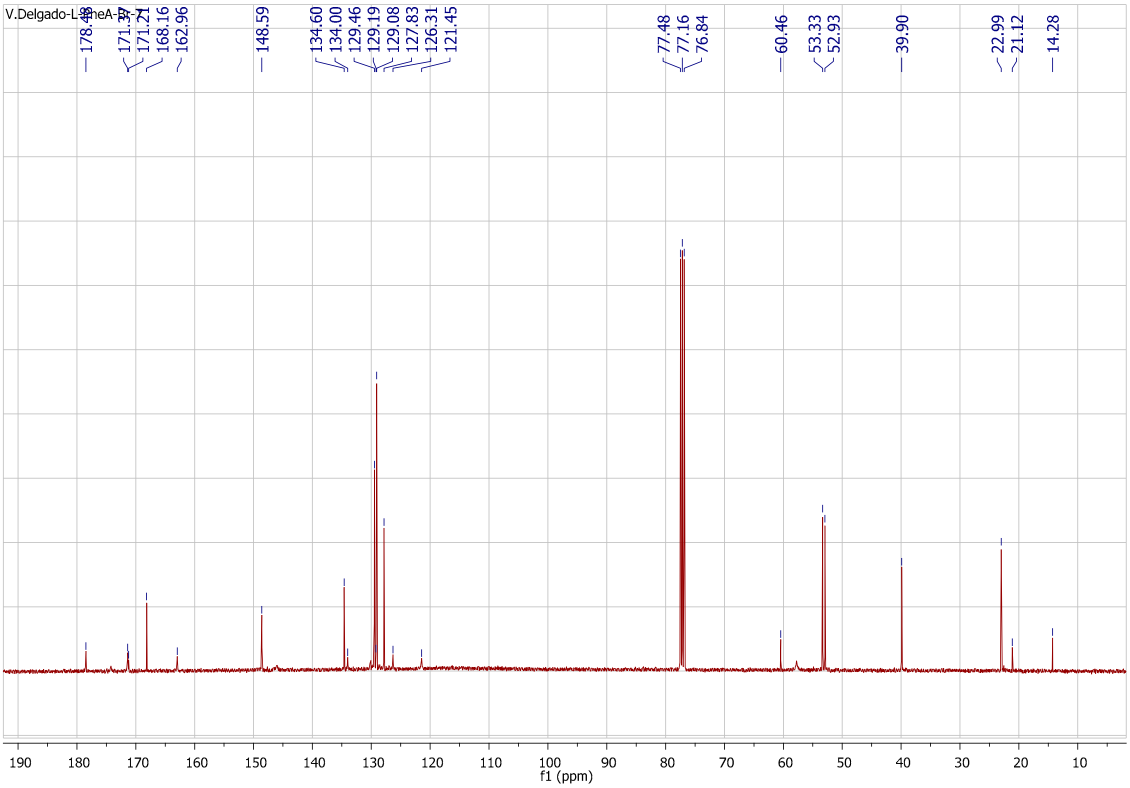

Figure S7. ^{1}H- and ^{13}C-NMR of compound **15**.
